# The Genome of the Blind Soil-Dwelling and Ancestrally Wingless Dipluran *Campodea augens*: A Key Reference Hexapod for Studying the Emergence of Insect Innovations

**DOI:** 10.1093/gbe/evz260

**Published:** 2019-12-03

**Authors:** Mosè Manni, Felipe A Simao, Hugh M Robertson, Marco A Gabaglio, Robert M Waterhouse, Bernhard Misof, Oliver Niehuis, Nikolaus U Szucsich, Evgeny M Zdobnov

**Affiliations:** 1 Department of Genetic Medicine and Development, Swiss Institute of Bioinformatics, University of Geneva Medical School, Switzerland; 2 Department of Entomology, University of Illinois at Urbana-Champaign; 3 Department of Ecology and Evolution, Swiss Institute of Bioinformatics, University of Lausanne, Switzerland; 4 Center for Molecular Biodiversity Research, Zoological Research Museum Alexander Koenig, Bonn, Germany; 5 Department of Evolutionary Biology and Ecology, Albert Ludwig University, Institute of Biology I (Zoology), Freiburg, Germany; 6 3rd Zoological Department, Natural History Museum Vienna, Vienna, Austria

**Keywords:** two-pronged bristletails, Entognatha, chemosensory genes, ionotropic receptors, gustatory receptors, photoreceptors

## Abstract

The dipluran two-pronged bristletail *Campodea augens* is a blind ancestrally wingless hexapod with the remarkable capacity to regenerate lost body appendages such as its long antennae. As sister group to Insecta (sensu stricto), Diplura are key to understanding the early evolution of hexapods and the origin and evolution of insects. Here we report the 1.2-Gb draft genome of *C. augens* and results from comparative genomic analyses with other arthropods. In *C. augens*, we uncovered the largest chemosensory gene repertoire of ionotropic receptors in the animal kingdom, a massive expansion that might compensate for the loss of vision. We found a paucity of photoreceptor genes mirroring at the genomic level the secondary loss of an ancestral external photoreceptor organ. Expansions of detoxification and carbohydrate metabolism gene families might reflect adaptations for foraging behavior, and duplicated apoptotic genes might underlie its high regenerative potential. The *C. augens* genome represents one of the key references for studying the emergence of genomic innovations in insects, the most diverse animal group, and opens up novel opportunities to study the under-explored biology of diplurans.

## Introduction

Diplura, also referred to as two-pronged bristletails, are ancestrally wingless hexapods. With a worldwide distribution, they constitute a ubiquitous component of soil ecosystems, occurring primarily in the litter layer and humid soils of forests and grasslands, but even colonizing deeper soil layers (Orgiazzi et al. 2016). Their soft, mostly unpigmented, and weakly sclerotized elongated bodies range up to 10 mm (Sendra et al. 2018). All diplurans are blind and possess long myocerate (bead-like) antennae, a pair of filamentous terminal appendages (cerci), as well as remnants of abdominal legs (styli) (Grimaldi 2010). Larval development is epimorphic (i.e., body segmentation is completed before hatching), and molting continues during the entire life span (Koch 2009), likely playing an important role in their remarkable capacity to regenerate lost body appendages (Maruzzo et al. 2005; Whalen and Sampedro 2010; Orgiazzi et al. 2016; Tyagi and Veer 2016). Fertilization of the diplurans’ eggs is indirect: Males produce and deposit spermatophores (sperm packets) that are subsequently collected by conspecific females (Koch 2009). Approximately 1,000 species of Diplura have been described and divided into 3 main groups: Campodeoidea with long segmented and filiform cerci, Japygoidea with unsegmented pincer-like cerci, and Projapygoidea with short cerci bearing spinning glands (Koch 2009). Although the latter two are predators and eat small arthropods, Campodeoidea, of which *Campodea augens* is a typical representative, are omnivores and are part of the decomposer soil community (Carpenter 1988; Lock et al. 2010). One distinctive feature of diplurans respect to insects (Insecta sensu stricto) is the position of the mouthparts that are hidden within head pouches (entognathous) (Böhm et al. 2012) like in Collembola (springtails) and Protura (coneheads), whereas in insects the mouthparts are exposed (ectognathous). However, many features present in diplurans have been retained in insects (sensu stricto), and phylogenetic and morphological studies suggested that Diplura likely represent the sister group of insects (Machida 2006; Sasaki et al. 2013; Misof et al. 2014), making Diplura a crucial reference taxon when studying the early evolution of insect genomes. Despite their evolutionary importance, diplurans have remained underexplored in particular at the genomic level, hampering a deeper understanding of the early evolution of hexapod genomes. Therefore, we sequenced and annotated the genome of *C. augens*, and present here the first analysis of a dipluran genome. For some of our analyses, we got permission to include another dipluran genome (*Catajapyx aquilonaris*) representing the lineage Japygoidea, which have been sequenced in the context of the i5k initiative (https://i5k.nal.usda.gov/; last accessed November 29, 2019). By analyzing and comparing *C. augens* genome with those of 12 other arthropods we found evidence for rapid gene family evolution in *C. augens*. For example, we uncovered a massive expansion of the ionotropic receptor gene family, which might compensate for the loss of vision. We also described expansions of gene families related to detoxification and carbohydrate metabolism, which might reflect adaptations of *C. augens*’ foraging behavior, and duplications of apoptotic genes, which could underlie its regenerative potential. Intriguingly, in the genome we discovered putative remnants of endogenous viral elements resembling negative single-stranded RNA viruses of the Orthomoxyviridae, a viral family that includes the Influenza viruses. With Diplura representing the sister clade to Insecta, the genome of *C. augens* serves as a key outgroup reference for studying the emergence of insect innovations, such as the insect chemosensory system, and opens up novel opportunities to study the underexplored biology of diplurans. 

## Materials and Methods 

### Sample Collection and Sequencing

*Campodea augens* samples were collected at Rekawinkel, Austria (48°11′06,68″N, 16°01′28,98″E) and determined based on the key of Palissa (1964) complemented by more recent taxonomic information (Voucher specimen IDs: NOaS 220-244/2019, preserved at the Natural History Museum Vienna). *Campodea augens* genome size was estimated to be ∼1.2 Gb by flow-through cytometry following the protocol given by DeSalle et al. (2005) using *Acheta domestica* as size standard (ca. 3.9 Gb). Two female adults were used for genome sequencing. Before DNA extraction, the individuals were carefully washed to remove any nontarget organisms that might adhere on the body surface. Genomic DNA was extracted using a Qiagen DNeasy Blood & Tissue kit (Qiagen, Hilden, Germany) and following the “insect” nucleic acid isolation protocol described by the manufacturer. Four Illumina paired-end (PE) sequencing libraries, 2 × 350- and 2 × 550-bp insert sizes, were constructed using Illumina’s TruSeq DNA Nano kit (Illumina, San Diego, CA) following the standard protocol. Four additional mate pair (MP) libraries (3-, 6-, 9-, and 12-kb insert sizes) were prepared using Illumina’s Nextera Mate Pair kit with size selection performed on precast E-gel (Life Technologies, Europe BV) 0.8% agarose gels. Libraries were sequenced on a HiSeq 2500 platform (Illumina) using a read-length configuration of 2 × 100 bp. All raw reads (around 2.1 billion in total) are deposited in the NCBI Sequence Read Archive (SRA) under the accession numbers SRX3424039–SRX3424046 (BioProject: PRJNA416902). 

### Genome Assembly

Low-quality reads and reads with adaptor and spacer sequences were trimmed with trimmomatic v0.36 (Bolger et al. 2014). The kmer content of reads from short-insert libraries was calculated using KmerGenie v1.7023 (Chikhi and Medvedev 2014). GenomeScope v1.0 (Vurture et al. 2017) was used to assess the heterozygosity level. All libraries were initially screened to detect reads derived from 16S genes of bacterial and archaeal species with the program parallel-meta v3 (Jing et al. 2017) using the “shotgun” option. Further screening of the reads was performed with Kraken (Wood and Salzberg 2014), using a set of custom databases representing full genomes of archaea, bacteria, fungi, nematodes, plants, protozoa, viruses, and worms. An initial draft assembly constructed from short-insert libraries using sparseassembler (Ye et al. 2012) was used for assessing the presence of contamination by Taxon-Annotated GC-Coverage plots as in Blobtools (Laetsch and Blaxter 2017). For taxonomic annotation of the draft contigs, results from MegaBLAST v2.3.0+ (Camacho et al. 2009) using the NCBI nucleotide database as well as Diamond v0.8.34.96 (Buchfink et al. 2015) with UniRef90 protein database (Suzek et al. 2015) were used as input for Blobtools (run using the “bestsumorder” rule). Read coverage for each contig was calculated by mapping the four libraries to the draft assembly using BWA v0.7.13 (Li and Durbin 2009). We further analyzed the assembly with PhylOligo (Mallet et al. 2017) using hierarchical DBSCAN clustering (phyloselect.py) and with Anvi’o (Eren et al. 2015) following the procedure shown in Delmont and Eren (2016) in order to visualize the assembly (only contigs >2.5 kb) along with GC content and mapping profiles to detect potential contaminants. In order to identify *C. augens* mitochondrial sequences, the draft contigs were compared with BLASTN (Camacho et al. 2009) to available mitochondrial genomes of two dipluran species (*Campodea fragilis* and *Campodea lubbocki*) (Podsiadlowski et al. 2006). The results of the abovementioned analyses were used to filter the raw reads: Contigs that showed a substantially different coverage/GC composition relative to that of the main cluster of contigs or hits to putative contaminants were used to filter the reads by mapping with BWA. Filtered short-insert libraries were then reassembled using the heterozygous-aware assembler Platanus v1.2.4 (Kajitani et al. 2014) with its default parameters. Redundans v0.13a (Pryszcz and Gabaldón 2016) was used to decrease the assembly redundancy, which occurs when heterozygous regions are assembled separately (Kelley and Salzberg 2010). Scaffolding of the contigs was performed with SSPACE v3.0 (Boetzer et al. 2011) using both short-insert and MP libraries. A final step of scaffolding was performed with AGOUTI v0.3.3 (Zhang et al. 2016) using *C. augens* transcripts obtained from reassembling raw reads (SRR921576) from whole-body RNA-seq data produced in the context of the 1KITE project (www.1kite.org; last accessed May 9, 2019). Scaffolds <1 kb were filtered out unless they showed a significant (e-value <1e−05) similarity to entries in the NCBI nonredundant (nr) database. To assess the quality of the assemblies and to monitor the improvement in assembly quality, each step was monitored with BUSCO v3.0.2 (Waterhouse et al. 2018), using the arthropoda_odb9 data set. The completeness and the accuracy of the genome assembly were further assessed by mapping the PE reads to the assembly using BWA and the assembled transcripts to the genome assembly using TopHat (Trapnell et al. 2009). This Whole Genome Shotgun project has been deposited at DDBJ/ENA/GenBank under the accession VUNT00000000. The version described in this paper is version VUNT01000000. *Campodea augens* mitochondrial genome was assembled separately using NOVOPlasty v2.6.7 (Dierckxsens et al. 2017), and annotated using MITOS (Bernt et al. 2013). The mitogenome sequence was deposited in GenBank under the accession number MN481418. The assembly and additional information such as the short (<1 kb) scaffolds can also be obtained from http://cegg.unige.ch/campodea_augens; last accessed November 29, 2019.

### Genome Annotation

A custom *C. augens* repeat library was constructed using RepeatModeler and RepeatClassifier (http://www.repeatmasker.org/; last accessed November 29, 2019). The species-specific repeats library and the RepBase (Bao et al. 2015) library (update 2 March 2017) were used to mask the genome with RepeatMasker v4.0.7 (http://www.repeatmasker.org; last accessed November 29, 2019). Automated annotation was performed with MAKER pipeline v2.31.8 (Holt and Yandell 2011) on the masked genomic scaffolds. Evidence-based gene structural annotations were inferred using eight arthropod proteomes obtained from OrthoDB v9 (Zdobnov et al. 2017), all entries from the SwissProt database (Boutet et al. 2016), and the *C. augens* transcripts obtained by reassembling raw reads (SRR921576) from whole-body RNA-seq data. MAKER was set to infer gene models from all evidence combined (not only transcripts), and gene predictions without transcript evidence were allowed. AUGUSTUS v3.2 (Stanke and Morgenstern 2005), trained with parameters obtained from *C. augens* single-copy genes identified with BUSCO (Seppey et al. 2019), was used for ab initio gene prediction.

The *C. augens* genome along with gene predictions, raw reads, and additional tracks were loaded into Apollo (Lee et al. 2013), which was used to visualize and manually curate gene models of families of interest. Chimeric gene models were split into individual genes, erroneously split gene models were joined, and exon–intron boundaries were corrected according to transcript evidence. This resulted in 23,992 gene models, which were assigned identifiers from CAUGE_000001 to CAUGE_023992 (where “CAUGE” stands for *Campodea AUGEns)*. Functional annotation of predicted protein-coding genes was performed using InterProScan for finding conserved domains. Additionally, proteins were searched against Uniref50 database (Suzek et al. 2015) and clustered in OrthoDB v10 (Kriventseva et al. 2019) for finding conserved functions.

To assign *C. augens* genes to orthologous groups (OGs), the predicted gene set was clustered with the gene sets of 169 arthropods in OrthoDB v10 database (Kriventseva et al. 2019). Subsequently, a phylogenomic analysis was conducted based on 371 single-copy genes shared among *C. augens* and 13 other arthropods including 1) insect species: *Acyrthosiphon pisum* (pea aphid), *Apis mellifera* (honey bee), *Calopteryx splendens* (banded demoiselle), *Danaus plexippus* (monarch butterfly), *Drosophila melanogaster* (fruit fly), *Pediculus humanus* (body louse), and *Tribolium castaneum* (red flour beetle); 2) representatives of ancestrally wingless hexapods: *Acerentomon* sp. (coneheads), *Catajapyx aquilonaris* (northern forcepstail), *Folsomia candida* (springtail), *Orchesella cincta* (springtail); and 3) two nonhexapod species: *Daphnia pulex* (water flea) and *Strigamia maritima* (centipede). No genome is available for the Protura order, but since this taxon is crucial in the context of our phylogenetic analysis, we reassembled the transcriptome of *Acerentomon* sp. from raw reads deposited at SRA (SRR921562) and used the resulting assembly as the source of phylogenomic markers. The *Acerentomon* sp. transcriptome was assembled using Trinity (Grabherr et al. 2011) with its default parameters. For extracting single-copy genes and performing phylogenomic analysis, we followed the pipeline described in Waterhouse et al. (2018). Briefly, MAFFT (Katoh and Standley 2013) was used for generating multiple sequence alignments of the amino acid sequences, alignments were then automatically trimmed with Trimal v1.2.59 (Capella-Gutiérrez et al. 2009), and RAxML v8 (Stamatakis 2014) was used for inferring a phylogenetic tree under the maximum likelihood method (supplementary note, Supplementary Material online). The resulting phylogenetic tree was viewed and annotated using the R package ggtree (Yu et al. 2017).

### Gene Family Evolution (Expansions and Contractions)

To study gene family evolution, we first analyzed expansions and contractions of gene families using the software Computational Analysis of gene Family Evolution (café) (Han et al. 2013) and OrthoDB clusters (Kriventseva et al. 2019). Furthermore, to detect major size changes in gene families between the dipluran *C. augens* and more recent lineages of hexapods, we also compared the gene counts of gene families in *C. augens* with the average and median of gene counts from the six species of Insecta included in the analysis. The clustering data at the level Arthropoda from OrthoDB were converted into CAFE input files, and CAFE v4.1 was used to calculate the average gene expansion/contraction rate and to identify gene families that have undergone significant size changes (*P *<* *0.01) across the phylogeny (number of gene gains and losses). The rooted phylogenetic tree required for the analysis was inferred with RAxML as described in the previous section, excluding data for the proturan *Acerentomon* sp., for which a genome is not yet available. The time-calibrated phylogeny was estimated using the r8s program (Sanderson 2003) using calibration points retrieved from timetree.org (Kumar et al. 2017) and from Misof et al. (2014). CAFE was run using the default *P*-value thresholds, and estimated rates of birth (λ) and death (μ) were calculated under a common lambda across the tree and additionally under different lambda for Collembola, Diplura, Insecta, and nonhexapod species.

Using OrthoDB clusters, we additionally identified gene families in *C. augens* significantly larger or smaller with respect to the average of counts in Insecta (s. str.) using a two-tailed Fisher’s exact test in R. The *P*-values were sequentially corrected using the Benjamini and Hochberg method (Benjamini and Hochberg 1995) in R, and only gene families with corrected *P*-values <0.01 were considered as significantly different.

We complemented the analysis of gene families by performing an overall protein domain analysis. We analyzed the proteomes of all 13 arthropod species included in the phylogenomic analysis using InterProScan (Jones et al. 2014) and searched for known Pfam protein domains, filtering out entries covering <75% of the corresponding Pfam hidden Markov model (HMM). Applying the same Fisher’s test as above, we identified protein domains with significantly larger counts in *C. augens* respect to the average found in the insect species.

### Identification and Phylogenetic Analysis of Endogenous Viral Elements

*Campodea augens* sequences with high identity with those of viral peptides were extracted and used to screen the GenBank nr database in a reciprocal TBLASTN search. Analyses of the putative insertion sites (i.e., raw read coverage of the sites and corresponding flanking regions, presence of host genes in the surrounding regions, and phylogenetic analyses) were performed to exclude the possibility of dealing with assembly artifacts. For the phylogenies, regions of putative endogenous viral elements were translated into amino acid sequences and individually aligned to amino acid sequences of the polymerase basic protein 1 (PB1) from orthomyxoviruses retrieved from GenBank database (supplementary table 13, Supplementary Material online). Alignments were generated with MAFFT (Katoh and Standley 2013), and the phylogenetic trees were constructed using the neighbor-joining method with a nonparametric bootstrap analysis (1,000 replicates). The resulting trees were visualized and annotated in Evolview (He et al. 2016).

### Indepth Analysis of Genes of Interest

The chemoreceptor genes in the GR and IR families were manually annotated. An attempt was made to build all models that include at least half of the coding region of related full-length IRs, including genes truncated due to problems with the genome assembly (sequence gaps or misassemblies indicated by single Ns) and pseudogenes. *Campodea augens* IRs were named in a series starting from Ir101 (Ir101–117 share a single intron) to avoid confusion with *D. melanogaster* genes, which were named after their cytological location and hence stopping with Ir100a. TBLASTN searches with representatives from insects, such as the damselfly *Calopteryx splendens* (Ioannidis et al. 2017), were used to identify genes using no filter for low complexity regions, word size of 2, and an E-value of 1,000. Models for the GRs and the intron-containing IRs were built in the Apollo genome browser (Lee et al. 2013) employing the automated models from MAKER and RNA-seq for support. The large set of intronless IR models and their many pseudogenes were built in a text editor, because pseudogene models are difficult to build in the Apollo browser. Encoded proteins were aligned with representatives from other species using ClustalX v2.0 (Larkin et al. 2007), and models refined in light of these alignments. The final alignments were trimmed using TrimAl v4.1 (Capella-Gutiérrez et al. 2009) with the “gappyout” option for the GRs and the “strictplus” option for the IRs, which have more length variation than the GRs. This analysis excludes the few available partial GR sequences from two orders of early diverging insects (Archaeognatha and Zygentoma) (Missbach et al. 2014). Unfortunately, the available draft genomes of these two orders are too fragmented to reliably annotate their GRs (Brand et al. 2018), and the GRs have been only partially annotated for one of the three draft genomes of Collembola (Wu et al. 2017). Given the phylogenetic distance of Diplura to Collembola and ancestrally wingless insects (i.e., Archaeognatha and Zygentoma), inclusion of their GRs is unlikely to change our assessment of the dipluran GR complement. Maximum likelihood phylogenetic analysis was performed using PhyML v3.0 (Guindon et al. 2010) at the PhyML webserver using the software’s default settings (http://www.atgc-montpellier.fr/phyml/; last accessed March 15, 2019). Trees were prepared using FigTree v1.4.2 (http://tree.bio.ed.ac.uk/software/figtree/; last accessed March 15, 2019). The Hox genes, photoreceptors and clock genes were searched with TBLASTN on the genome assembly using a set of known proteins and by InterProScan results. Gene models were checked and curated in the Apollo browser. For candidate opsins, the *C. augens* gene set and whole genome were scanned using 16 reference opsins (Hering and Mayer 2014) with BLASTP and TBLASTN, respectively, matches with an e-value <1e−4 were retained as candidate opsins. Additionally, HMM profiles created for the nine major opsin clades (cnidopsins, vertebrate c-opsins, pteropsins, Group 4 opsins, arthropsins, melanopsins, nonarthropod r-opsins, arthropod visual opsins, and onychopsins) (Hering et al. 2012) were used to scan the *C. augens* gene set.

## Results and Discussion

### The *C. augens* Genome

**Fig. 1.**
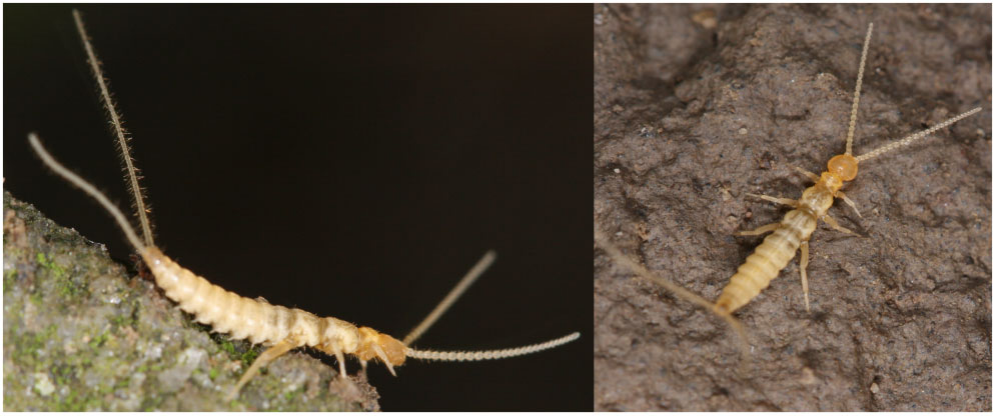
—Lateral and dorsal view of *Campodea augens*.

We assembled the genome of the dipluran *C.augens* (fig. 1; supplementary fig. 1, Supplementary Material online) using 120 Gb of Illumina PE reads data, representing ∼100× coverage of the genome, and additional 90 Gb of mate-pair reads (supplementary fig. 2 and table 1, Supplementary Material online). The inferred draft assembly spans 1.13 Gb, which is close to the genome size of 1.2 Gb estimated via flow cytometry, indicating that most of the genome is likely present in the assembly (supplementary note and table 2, Supplementary Material online). The high level of completeness was also confirmed with BUSCO (Waterhouse et al. 2018) as detailed below. Although genomes of many hexapod groups have recently been sequenced, only few are from ancestrally wingless hexapods: Four collembolans (*Folsomia candida*, *Orchesella cincta, Holacanthella duospinosa*, and *Sinella curviseta*) (Faddeeva-Vakhrusheva et al. 2016, 2017; Wu et al. 2017; Zhang et al. 2019), whose genomes have been published; and a dipluran (*Catajapyx aquilonaris*) which have been sequenced in the context of the i5k initiative (https://i5k.nal.usda.gov/; last accessed November 29, 2019). Currently, there are no sequenced genomes of proturans.

**Table 1:** Counts of Protein Pfam Domains Associated With Detoxification Enzymes in *Campodea augens* and 12 Other Arthropods

				Collembola		Diplura		Insecta						
Gene Family	pfamID	Smar	Dpul	Fcan	Ocin	Caqu	*Caug*	Cspl	Apis	Phum	Amel	Tcas	Dple	Dmel
ABC transporter (ABC)	PF00005	74	70	140	113	59	229	53	126	39	51	77	60	56
	PF00664	28	16	50	44	22	56	23	31	12	16	42	28	22
	PF06472	3	3	3	3	3	5	3	2	2	2	2	2	2
Carboxylesterase (CCE)	PF00135	21	40	120	110	32	147	47	48	22	26	51	57	35
Cytochrome P450 (CYP)	PF00067	48	75	214	260	71	202	90	82	39	46	125	73	89
Glutathione S-transferase (GST)	PF00043	4	6	6	4	7	25	8	19	7	4	22	13	25
	PF02798	6	18	52	32	5	22	11	12	6	6	12	9	15
	PF13417	4	8	11	9	5	18	5	17	6	5	22	14	24
UDP glycosyltransferase (UGT)	PF00201	16	24	51	57	8	104	18	72	4	12	40	36	35
Total		204	260	647	632	212	808	258	409	137	168	393	292	303

Dmel, *Drosophila melanogaster*; Dple, *Danaus plexippus*; Tcas, *Tribolium castaneum*; Amel, *Apis mellifera*; Phum, *Pediculus humanus*; Apis, *Acyrthosiphon pisum*; Caug, *Campodea augens*; Cspl, *Calopteryx splendens*; Caqu, *Catajapyx aquilonaris*; Fcan, *Folsomia candida*; Ocin, *Orchesella cincta*; Dpul, *Daphnia pulex*; Smar, *Strigamia maritima*.

Genomes of ancestrally wingless hexapods have proved difficult to sequence, as they are often large (as in the case of *C. augens*), and because many species are typically difficult to rear. Thus, it is almost impossible to reduce natural heterozygosity (e.g., via inbreeding experiments), which potentially compromises genome assembly efforts (Richards and MuraLi 2015). Our *C. augens* assembly is much larger than the assemblies of *C. aquilonaris* (312 Mb) and of the four collembolans (221–381 Mb) (supplementary fig. 3 and table 2, Supplementary Material online). The large genome size of *C. augens* is likely explained by the proliferation of repeats, which account for ∼46% of the assembly (supplementary table 3, Supplementary Material online). Most of these repeats are unclassified and represent 33% (376 Mb) of the assembly. Among the identified repetitive elements, the *hAT* superfamily is the most abundant DNA transposon and accounts for 5.9% of the genome sequence (supplementary table 3, Supplementary Material online). The high level of repeats posed a challenge for genome assembly, resulting in a total of 18,765 scaffolds with N_50_ values for contigs and scaffolds at 33 and 235 kb, respectively (supplementary fig. 3 and table 2, Supplementary Material online). The annotated gene set of *C. augens* comprises 23,978 predicted protein-coding genes and exceeds in number that of *C. aquilonaris* (10,901), however, it is comparable to that of *F. candida* (22,100) and that of *O. cincta* (20,247) (supplementary table 4, Supplementary Material online). More than 90% of the gene models have support from *C. augens* transcripts. Approximately 74% of the predicted proteins show a significant amino acid similarity (BLASTP e-value <1e−10) to existing entries in the Uniref50 database, whereas ∼95% of the proteins contain a domain assigned with InterProScan, providing high confidence in the accuracy of annotations. Assessments with Benchmarking Universal Single-Copy Orthologs (BUSCOs) (Waterhouse et al. 2018) identified 97.0–97.6% (90.4–91.8% complete) of the 1,066 BUSCOs expected to be conserved in arthropods, indicating high completeness of the assembly and gene models (supplementary fig. 4 and table 5, Supplementary Material online). The completeness of the gene set is additionally indicated by the successful identification of all *Hox* genes known from *F. candida* (*lab, hox3, pb, ftz, src, dfd, antp, ubx, abd-A*, and *abd-B*). In *C. augens*, however, these genes are distributed across five scaffolds (supplementary fig. 5, Supplementary Material online) spanning more than 1 Mb.

**Fig. 2.**
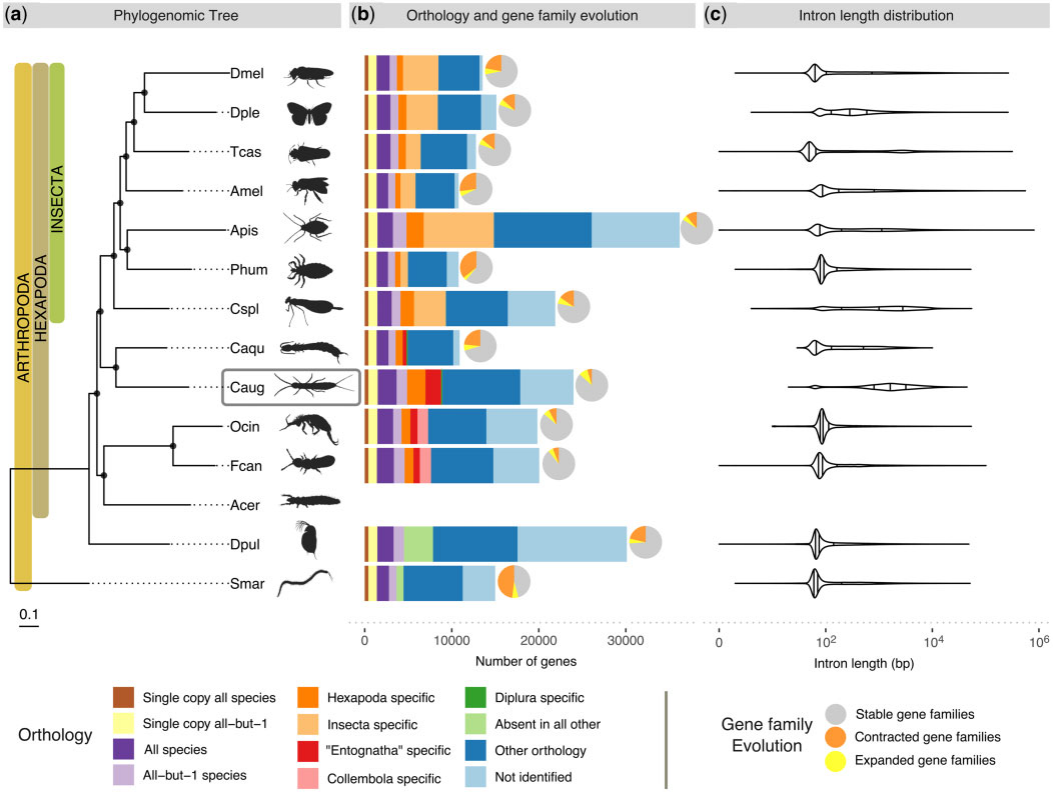
—Species phylogeny, orthology, gene family expansions/contractions, and intron length distributions of *Campodea augens* and 13 other arthropod species. (*a*) Phylogenomic tree of 14 arthropod species, including ancestrally wingless hexapods: 2 diplurans, 2 collembolans, and 1 proturan (data for proturan were obtained from transcriptome). The maximum likelihood phylogeny was estimated from the aligned protein sequences of 371 single-copy orthologs, using the centipede, *Strigamia maritima*, as the outgroup. Branch lengths represent substitutions per site. Black circles on nodes indicate bootstrap support >0.95. Dmel, *Drosophila melanogaster* (fruit fly); Dple, *Danaus plexippus* (monarch butterfly); Tcas, *Tribolium castaneum* (red flour beetle); Amel, *Apis mellifera* (honey bee); Phum, *Pediculus humanus* (body louse); Apis, *Acyrthosiphon pisum* (pea aphid); Cspl, *Calopteryx splendens* (banded demoiselle); Caqu, *Catajapyx aquilonaris* (northern forcepstail); Acer, *Acerentomon* sp. (coneheads); Fcan, *Folsomia candida* (springtail); Ocin, *Orchesella cincta* (springtail); Dpul, *Daphnia pulex* (water flea); Smar, *Strigamia marítima* (centipede). (*b*) Bars: Total gene counts per species grouped into the following categories: Single-copy orthologs in all 13 species or in all except one species, present in all or all except one species allowing for gene duplications, lineage-specific orthologs (Hexapoda, “Entognatha”, Collembola, Diplura, and Insecta), genes present in only 1 species (absent in other species), genes with all other ortholog relationships, and with no identifiable orthologs; pie charts: Proportions of species-specific gene family expansions (yellow), contractions (orange), and stable gene families (gray) as estimated using CAFE (these proportions include the number of nonsignificant expansions/contractions, see text and supplementary fig. 8, Supplementary Material online for more details). (*c*) Violin plots of intron length distributions of the corresponding species in the phylogeny. Vertical bars correspond to median, lower, and upper quartiles. Silhouette images were obtained from PhyloPic (http://phylopic.org; last accessed March 10, 2019). All are under public domain except: *Calopteryx splendens* by Maxime Dahirel; Protura, *Orchesella* springtail, and *Strigamia* centipede by Birgit Lang; all licensed under the Creative Commons Attribution 3.0 Unported license (http://creativecommons.org/licenses/by/3.0/; last accessed March 10, 2019); and *Acyrthosiphon pisum* by Joseph Hughes licensed under Creative Commons Attribution NonCommercial-ShareAlike 3.0 Unported licence (https://creativecommons.org/licenses/by-nc-sa/3.0/; last accessed March 10, 2019).

The number of introns/exons per gene and exon lengths in *C. augens* are similar to those of *C. aquilonaris* and those of collembolans (supplementary table 4, Supplementary Material online). However, the median size of the gene models in *C. augens* is roughly four times longer than in *C. aquilonaris* and three to five times longer than in collembolans (supplementary table 4 and fig. 6, Supplementary Material online). This is mostly due to longer introns in the genome of *C. augens* (fig. 2; supplementary table 4 and fig. 6, Supplementary Material online). Indeed, introns remarkably span 27% of the *C. augens* genome, accounting for a total of 306 Mb, which is equivalent in size to the entire *C. aquilonaris* genome*.* It has been proposed that longer introns are associated with an increased metabolic cost (Vinogradov 1999), so strong selection against long introns is expected. Various factors could have promoted the evolution of large intron sizes in *C. augens*, ranging from drift to low-genome-wide recombination rates, and remain to be studied*.* The differences in gene content, genome size, and intron sizes between C*. augens* and *C. aquilonaris* suggest that these two dipluran genomes have differentiated greatly from each other, likely through the spread of repetitive elements in *C. augens*.

### Phylogenomics and Orthology

Although the monophyly of Hexapoda is now well supported (Grimaldi 2010; Sasaki et al. 2013; Misof et al. 2014), the relationships among Protura, Collembola, and Diplura (traditionally grouped as “Entognatha”) and their placements with respect to ectognathous hexapods (Insecta) have been much debated. The following hypotheses exist regarding the phylogenetic position of Diplura: 1) Diplura as sister group of Protura and Collembola (Entognatha hypothesis) (von Reumont et al. 2009); 2) Diplura as sister group of Protura (Nonoculata hypothesis) (Luan et al. 2005; Meusemann et al. 2010; Andrew 2011); and 3) Diplura as sister group of Insecta *s. str.* (Kukalová-Peck 1998; Misof et al. 2014) with “Entognatha” being paraphyletic. Although the fossil record of diplurans is poor, with the oldest known fossil from Lower Cretaceous deposits of Brazil (Wilson and Martill 2001), they probably originated in the Early Devonian (Misof et al. 2014). Phylogenomic analysis employing a supermatrix of 358 concatenated single-copy protein-coding genes of 14 arthropods support the idea of “Enthognatha” being paraphyletic and Diplura representing the sister group of Insecta (fig. 2a). This result is in agreement with previous phylogenetic studies that analyzed phylogenomic data (Misof et al. 2014), Sanger-sequenced nuclear-encoded protein-coding genes (Regier et al. 2004), or studied fossil (Kukalová-Peck 1987), morphological (Giribet et al. 2004), and comparative embryological (Ikeda and Machida 2001; Tomizuka and Machida 2015) evidence. However, more extensive sampling of genomes of Collembola, Diplura, and Protura is desirable to improve confidence in the inferred phylogenetic relationships.

Results from analyzing gene orthology via clustering the *C. augens* gene set with genes of 169 other arthropods are available from OrthoDB v10 (Kriventseva et al. 2019) (https://www.orthodb.org/; last accessed November 29, 2019). Analysis of the full set of OGs identified 19,063 *C. augens* genes that have orthologs in at least one other arthropod genome included in OrthoDB. Of these, 17,871 genes have orthologs in at least one of the 12 genomes under consideration in figure 2. In contrast, 4,929 *C. augens* genes have no identifiable orthology relationships with genes of other arthropods (and 6,121 genes when only considering species in fig. 2), and similarly for the two collembolans. The median protein length of these predicted genes is 209 amino acids, and some are likely to be gene fragments. Totally, 3,008 and 3,896 of them have at least 1 hit to a PFAM domain and at least 1 hit to another protein from the rest of the classified *C. augens* proteome (e-value <1e−05), respectively. Sequencing the genomes of additional ancestrally wingless hexapods and closely related outgroups (e.g., Remipedia) is required to reveal what fraction of these genes is indeed unique to *C. augens* or Diplura, or whether they evolved earlier in the history of Arthropoda.

### Gene Family Evolution

To examine in detail the most dramatic changes in the gene repertoires among ancestrally wingless hexapods, we modeled gene family evolutionary dynamics using the CAFE approach (Han et al. 2013). We detected significant (*P *<* *0.01) gene copy-number changes in OGs on both the branches leading to Diplura (19 expansions and 0 contractions) and to Collembola (45 expansions and 7 contractions) (supplementary fig. 8 and table 8, Supplementary Material online). The number of species-specific significant expansions and contractions varied within ancestrally wingless hexapods (*F. candida*: +49/−12; *O. cincta*: +28/−9; *C. aquilonaris*: +6/−30), with *C. augens* displaying the largest number of expansions and the smallest number of contractions (+90/−1) (fig. 2b). Among the expanded gene families in *C. augens* there are families of chemosensory receptors, specifically gustatory (GRs), and ionotropic receptors (IRs). A detailed analysis of these is presented below, where we report a massive expansion of IR gene family that accounts for ∼8% of the entire gene set content. Other expanded gene families detected with CAFE are involved in detoxification and sugar metabolism/transport, which may be related to the foraging ecology of *C. augens*, and in apoptosis, which may play a role in the high regeneration capacity of *C. augens* (supplementary tables 8, 9 and fig. 9, Supplementary Material online).

### The Gene Repertoire of *C. augens* Mirrors the Secondary Loss of an Ancestral External Photoreceptor Organ

**Fig. 3.**
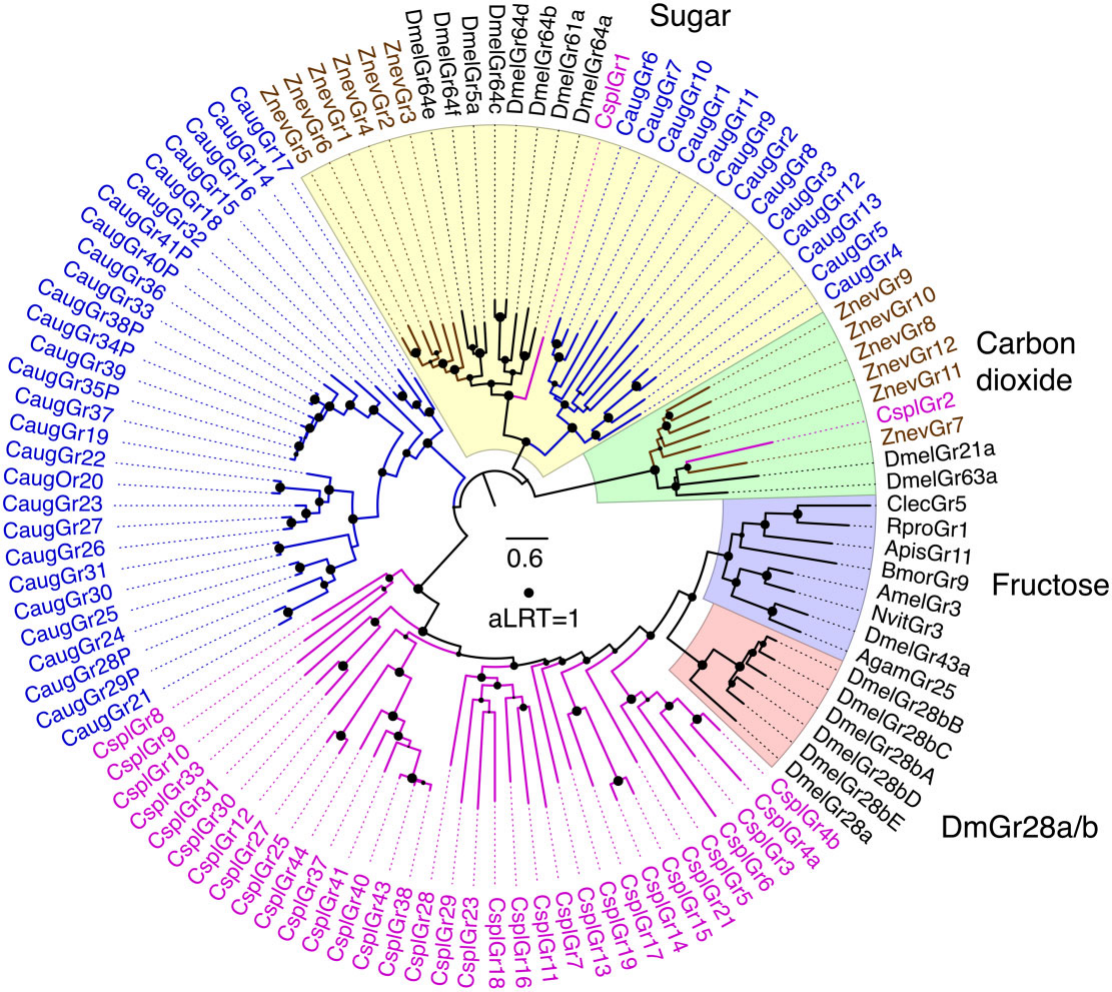
—Phylogenetic relationships of the *Campodea augens* GRs with representatives from other insects. The sugar and carbon dioxide receptor clades were defined as the outgroup based on their location in trees of the entire animal GR/GRL family (Robertson 2015). Major subfamilies or lineages are highlighted by colors and indicated outside the circle. *Campodea augens* GRs are colored blue. Representative GRs from other insects include the damselfly *C. splendens* (Cspl, purple) (Ioannidis et al. 2017), dampwood termite *Z. nevadensis* (Znev, brown) (Terrapon et al. 2014), and selected endopterygotes in black. The scale bar indicates substitutions per site and the size of the filled circles on nodes indicates approximate Likelihood Ratio Tests (aLRT) from PhyML v3.0 from 0 to 1.

The lack of external eyes and ocelli (simple eyes) in diplurans is presumably a consequence of degenerative processes of tissues that are functionless in dark soil environments. The eyeless collembolan *F. candida* detects both ultraviolet (UV) and white light (Fox et al. 2007; Gallardo Ruiz et al. 2017), and it has been hypothesized that the light avoidance behavior of *F. candida* is mediated by nonocular photoreceptors (Fox et al. 2007). Campodeids also avoid light and may sense it over their body surfaces (George 1963) having a thin and weakly sclerotized cuticle. A similar mechanism of internal photoreceptor cells or organelles might apply to *C. augens*. This kind of light-sensitivity might be sufficient for organisms that require light perception, but not spatial light resolution. However, it cannot be excluded that other environmental triggers, such as air temperature or relative humidity, play a role in the observed light-avoidance behavior of *C. augens* (George 1963). We searched the *C. augens* gene set for known photoreceptor genes, such as opsins, which in complex with molecular chromophores are sensitive to specific wavelengths of light. Although we found 149 G-protein-coupled receptor rhodopsin-like domains (Pfam PF00001), none of the corresponding proteins belong to the opsin subfamily. Using both profile HMMs of known opsins and TBLASTN searches of the genome, we found two fragments of putative opsins with best-hits to UV-sensitive opsins. One fragment contains the opsin retinal-binding site (prosite identifier: PS00238). Whether the opsin fragments are part of functional genes and play a role in the reported light avoidance behavior of diplurans awaits further investigation. In flies, the Gr28b set of receptors includes some involved in perception of light and heat (Xiang et al. 2010; Ni et al. 2013), and it is possible that in the absence of functional opsins, relatives of these GRs might confer light sensitivity to *Campodea* and other diplurans. We did not identify clear relatives of the DmGr28a/b lineage in the genomes of *C. augens* or that of *C. aquilonaris* (fig. 3), however, it is possible that other GRs have evolved this function in diplurans. Furthermore, we did not find DNA photolyases, which would repair DNA damage induced by UV light (supplementary table 10, Supplementary Material online), or cryptochrome proteins, which are components of the arthropod central circadian clock. DNA photolyases or cryptochromes were not found in the centipede *Strigamia maritima* (Chipman et al. 2014) and appear to be absent also in the dipluran *C. aquilonaris* and the collembolan *F. candida*, all three of which are eyeless. Intriguingly, the collembolan *O. cincta*, which possesses simple eyes, has DNA photolyases (supplementary table 10, Supplementary Material online). The loss of cryptochromes and DNA photolyases thus appears to be a common feature among blind arthropods with a subterranean lifestyle. With the paucity of photoreceptor genes, the gene repertoire of *C. augens* thus mirrors at the genomic level the secondary loss of the ancestral external photoreceptor organ. The absence of cryptochromes also calls into question the presence of the canonical circadian clock system, which in most organisms regulates various behaviors, biochemical and physiological functions, and which is set by light. Searches for components of the major regulatory feedback loop of the arthropod circadian clock (e.g., *clock*, *cycle*, *jetlag, period*, and *timeless*) revealed only partial homologs of *timeless* and *clock*. The absence of cryptochromes and of other known circadian clock genes such as *cycle*, *jetlag*, and *period* may indicate that *C. augens* has not maintained canonical circadian rhythms and thus possibly retained light-sensitivity primarily for orientation and habitat choice.

### GRs and the Massive Expansion of the IR Family Might Compensate for the Loss of Vision

**Fig. 4.**
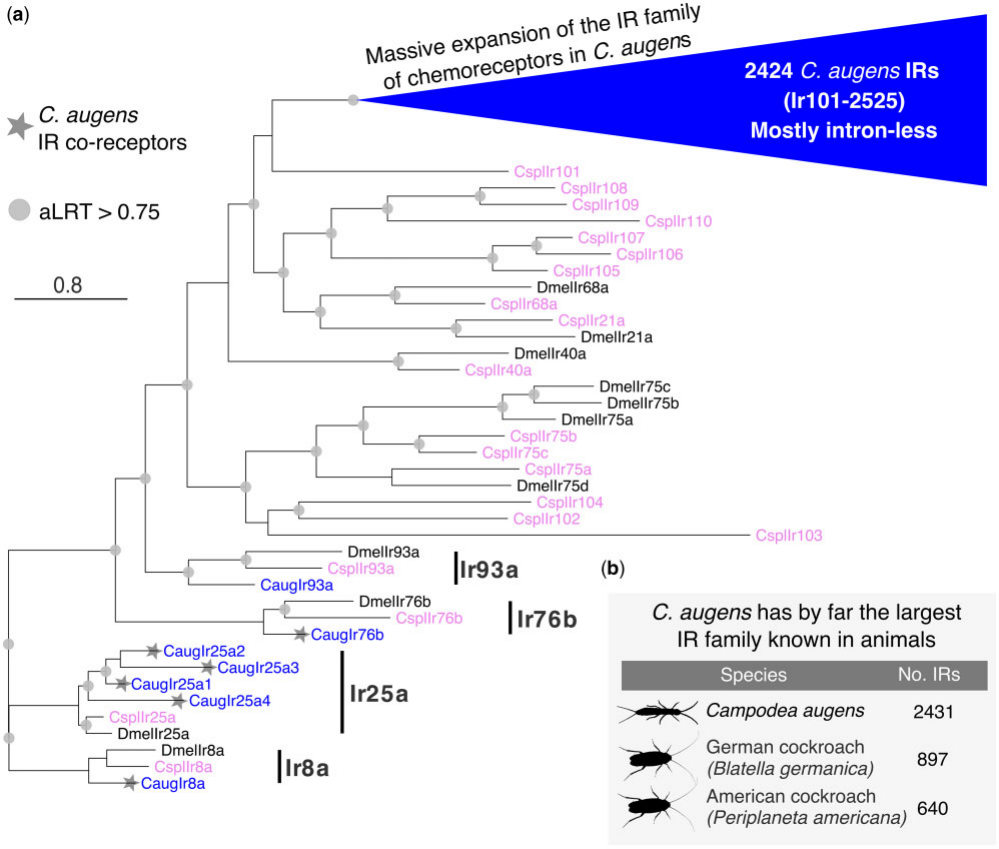
—Phylogenetic tree of the *Campodea augens* IR family. (*a*) The tree was rooted by declaring the Ir8a and 25a lineages as the outgroup, based on their basal positions within larger trees including the ionotropic glutamate receptors from which the IRs evolved. The blue triangle represents the massive expansion of *C. augens* IR family. Most of these 2424 IRs are intron-less, except for five lineages that have idiosyncratically gained introns (see supplementary fig. 10, Supplementary Material online for details). The *C. augens* (Caug) proteins are in blue, the *Drosophila melanogaster* (Dmel) proteins for the seven conserved IRs with orthologs in *C. augens*, as well as the Ir75 clade, are colored black, whereas the *Calopteryx splendens* (Cspl) proteins are colored purple. The four conserved lineages are marked with a black bar. *Campodea augens* IR coreceptors are marked with a star. The scale bar indicates substitutions per site. Gray circles indicate nodes with an approximate Likelihood-Ratio Test (aLRT) >0.75. (*b*) Table displaying the animal species with the largest repertoire of IRs. *Campodea augens* possesses by far the largest IR family known in animals to date. Silhouette images were obtained from PhyloPic (http://phylopic.org; last accessed March 10, 2019). All are under public domain.

Antennae are one of the most important olfactory organs of insects, and *C. augens* possesses one pair of long myocerate (bead-like) antennae that contain muscles protruding from the head and innervated on each side, allowing controlled movements of the antennae (Böhm et al. 2012). In the central nervous system, *C. augens* possesses two mushroom bodies and two different types of putative olfactory glomeruli: Four to five large and elongated ventral glomeruli and a set of small spheroidal dorsal glomeruli (Böhm et al. 2012) innervated from the antennae. These anatomical features suggest an advanced olfactory system in Diplura (Böhm et al. 2012). In fact, being blind, diplurans such as *C. augens* must depend largely on their senses of smell, taste, and touch to negotiate their environments, as indicated by their continual use of their long antennae while moving around. Given the likely sister group relationship to Insecta, Diplura represent a crucial taxon to understand the evolutionary origin of the chemosensory system in insects. However, studies of the genomic chemosensory amenities of Diplura are scarce. Brand et al. (2018) noted that the sequenced genome of the dipluran *C. aquilonaris* contains no odorant receptor (OR) gene family members. We likewise were unable to identify ORs or the OR coreceptor (Orco) in the *C. augens* genome. Along with the absence of ORs in three collembolan genomes (Wu et al. 2017), this observation corroborates the conclusion by Brand et al. (2018) that the OR gene family evolved in the stem lineage of Insecta. Instead, manual curation of the GR and IR gene families highlighted by the CAFE and domain analyses identified a modestly sized family of 41 GRs and a hugely expanded family of 2,431 IRs. This large repertoire of chemosensory genes likely provides *C. augens* with a sophisticated chemosensory system. Seven of the GRs are clear pseudogenes, and several gene fragments indicate additional pseudogene remnants. The most prominent subfamilies of arthropod GRs are the sugar receptors, which are also present in crustaceans such as *Daphnia pulex* (Peñalva-Arana et al. 2009) and *Hyalella azteca* (Poynton et al. 2018), and the carbon dioxide receptors (and their relatives), which have been found in early divergent insect lineages, such as Odonata (Ioannidis et al. 2017; Robertson 2019). *Campodeaaugens* has 13 candidate sugar receptors clustering with insect representatives of this subfamily (fig. 3) and sharing a glutamic acid immediately after the TY in the conserved TYhhhhhQF motif in the seventh transmembrane domain (where h is usually a hydrophobic amino acid). In contrast, there is no evidence for the presence of the carbon dioxide receptor subfamily or of a distinctive lineage of fructose receptors, both found in insect genomes (fig. 3). The remaining 28 GRs cluster together, with no close relatives in insects and by analogy with those of insects might include receptors for various “bitter” plant compounds and for cuticular hydrocarbons (Robertson 2019). The remarkably large set of 2,431 *C. augens* IRs contains representatives of each of the 3 conserved coreceptors, named after their *Drosophila melanogaster* orthologs (Ir8a, 25a, and 76b), with duplications resulting in four copies (paralogs) of Ir25a (we also detected fragments of a possibly fifth copy). Most insects have an additional set of single-copy IRs (Ir21a, 40a, 68a, and 93a) involved in the perception of temperature and humidity (Knecht et al. 2017), but only Ir93a was identified in *C. augens*. Intriguingly, Ir93a is also the only one of these four IRs to have been identified in several other arthropods groups, such as ticks, mites, centipedes, and crustaceans (Robertson 2019). However, the copepod *Eurytemora affinis* seems to encode a distant relative of Ir21 (Eyun et al. 2017; Poynton et al. 2018), implying that Ir21a was already present in the stem group of Hexapoda and has likely been lost from *C. augens* and other arthropods. No relatives of other IR lineages that are widely present in insects, for example, the Ir75 clade involved in perception of diverse acids (Prieto-Godino et al. 2017), seem to be present in the *C. augens* genome (fig. 4; supplementary fig. 10, Supplementary Material online). On the other hand, we found an extraordinary expansion of 2,424 mostly intronless IR genes, named from Ir101 to Ir2525, 901 of which are pseudogenes (defined as encoding at least 50% length of related IRs, there being innumerable additional shorter gene fragments). The vast majority of these genes are intronless in their coding regions, however, there are several lineages with one or two introns interrupting the coding region, all apparently gained independently from intronless ancestors, something previously observed in several insects, for example, the cockroach *Blatella germanica* (Robertson et al. 2018) in which the IR family expansion clearly represents an independent event from the expansion observed in *C. augens*. Thus Ir101–118 have a phase-2 intron about one-third into the coding region, although the divergent first exon was not identified for some of them. Divergent subfamilies of these IRs were discovered by weak matches in TBLASTN searches, so additional intron-containing genes were only detected toward the end of this iterative search process (because matches are weaker when the coding region is split by an intron). Thus Ir2291–2334 also have a phase-2 intron roughly one-third along. Ir2335–2371 have a phase-0 intron near the middle of the coding region, as do Ir2372–2413, but in a different location roughly one-third along. Finally, Ir2497–2523 have a phase-0 intron followed by a phase-2 intron. All of these six introns are not only in two different phases and are in different locations in the coding sequences, but as shown in supplementary figure 10, Supplementary Material online each is in a distinct gene lineage, so these six introns have been gained independently, and somewhat surprisingly have never been lost from any genes within each of these lineages. These IR genes exhibit many of the traits common to highly expanded gene families like these chemoreceptors in other animals, including some large arrays, albeit commonly with genes on both strands, implying considerable short-range inversions from an original tandem-orientation resulting from duplication by unequal crossing-over. Occasionally, a complicated pattern was noticed in which several scaffolds had comparable suites of otherwise distantly related genes in them, suggesting that segmental duplications have also played a part in the expansion of this gene family and hence the genome. Nevertheless, vast numbers are present as singletons in a scaffold, or far removed from others in a large scaffold, so there has been enormous genomic flux in this genome moving even individual genes around. These intronless and divergent IRs consist of numerous gene lineage expansions, many of which are very recent, as indicated by short branches, and are replete with pseudogenes and fragments, indicating rapid gene family turnover of the IRs in *C. augens* (fig. 4; supplementary fig. 10, Supplementary Material online). Large gene families typically have numerous pseudogenes and indeed at least 901 (37%) are pseudogenes, and as noted above there are innumerable apparently pseudogenic fragments. This is near the top end for percentage of chemoreceptor pseudogenes in arthropods (Robertson 2019), but less than some mammals, with over 50% pseudogenes being common (Niimura 2014). These pseudogenes are distributed throughout the family (supplementary fig. S10, Supplementary Material online), although there is a tendency for the most highly and recently expanded lineages to have the highest frequencies of pseudogenes, for example, in the bottom half of this circular tree. This is visually conspicuous in the tree because these pseudogene names have a P at the end, making them longer than the related intact gene names. The numbers of obvious pseudogenizing mutations (stop codons, frameshifts, and large deletions) were noted for each pseudogene and a histogram (supplementary fig. 11, Supplementary Material online) reveals that the majority have single mutations, a pattern seen previously for the cockroach IRs (Robertson et al. 2018), consistent with the young nature of most of these pseudogenes in recent gene lineage expansions. Presumably, most older pseudogenes also eventually suffer major deletions that reduce them to the innumerable fragments observed.

The total of 2,472 chemoreceptor genes is comparable to the largest numbers seen in some mammals, such as 2,294 ORs in the cow, 2,658 in the horse, and 4,267 in the elephant (Niimura 2014), although these counts do not include their vomeronasal or taste receptors, and is far larger than any chemoreceptor repertoire known in other arthropods, the two highest so far being 825 in the spider mite *Tetranychus urticae* (Ngoc et al. 2016) and 1,576 in *B. germanica* (Robertson et al. 2018). This remarkable expansion of the IR family exceeds even those found in the genomes of cockroaches with 640 and 897 (Li et al. 2018; Robertson et al. 2018), making this the largest IR family known in animals. This massive expansion of the IR family of chemoreceptors in this genome presumably reflects the dependence of this dipluran on its chemical senses of both smell and taste.

### Expansion of Gene Families Related to Xenobiotic Detoxification and Apoptosis

Analysis of the gene sets revealed that *C. augens* possesses the largest set of enzymes related to detoxification (808 genes) among the 13 species examined including representatives of main hexapod orders (table 1). These families were found to be expanded and include ATP-binding cassette transporters (ABC transporters), carboxylesterases (CEs), cytochrome P450s (CYPs), UDP-glucoronosyl/glucosyl transferases (UGTs), and Glutathione S-transferases (GSTs) (supplementary tables 8 and 9, Supplementary Material online). *Campodea augens* has the largest set of ABC transporters, 290 in total, thus 1.5 times as many as the second largest set of ABC transporters identified in the collembolan *F. candida* (193 in total). Likewise, carboxylesterases are more abundant in *C. augens* (147) with repertoires in collembolans being nearly as large (*F. candida*, 120 genes; *O. cincta*, 110 genes). Cytochrome P450 domains in *C. augens* (202 in total) are similar in number to those in collembolans (*F. candida*, 214; *O. cincta*, 260), but they are much higher than in all other arthropods examined. GST counts are within the ranges observed in the other species, whereas UGTs are again most abundant in *C. augens* (104 in total), double to those in collembolans (51 and 57, respectively) and 10 times as many than in the closely related dipluran *C. aquilonaris* (8 in total). Several of these genes are clustered in the *C. augens* genome, for example, sets of five P450s and four GSTs on scaffold_1992 and a set of six UGTs on scaffold_1829 (supplementary fig. 11, Supplementary Material online). As hypothesized for the two collembolans (Faddeeva-Vakhrusheva et al. 2016, 2017), the large repertoire of detoxification enzymes in *C. augens* might reflect adaptations to living in soil environments. The strikingly lower counts of detoxification genes in *C. aquilonaris* (212 in total), which also lives in the soil, may reflect its feeding behavior, which is different from that of *C. augens*. Although *C. augens* and the two collembolans are herbivorous or detritivorous, *C. aquilonaris* is a predator, feeding on small arthropods. Thus, *C. aquilonaris* might not have the same detoxification needs as *C. augens*. The latter likely has to cope with ingesting xenobiotic compounds that persist in decaying organic matter, such as plant antiherbivory toxins, lignocellulose by-products, and feeding deterrents.

In contrast to other hexapods, diplurans have the remarkable capacity to regenerate lost body appendages (antennae, cerci, and legs) over a series of molts, even as adults (Lawrence 1953; Conde 1955; Maruzzo et al. 2005; Whalen and Sampedro 2010; Tyagi and Veer 2016). The genetic amenities to facilitate these adaptations have not been investigated to date. However, candidate genes related to regenerative capacities of tissues have been studied in model organisms (Bergmann and Steller 2010). For example, caspases are protease enzymes involved in programed cell death that also play a role in tissue regeneration and differentiation in some animal models (Bergmann and Steller 2010; Sun and Irvine 2014), including *D. melanogaster*. Apoptosis and caspase activity have been detected in regenerating tissue (Boland et al. 2013; Shalini et al. 2015). In *C. augens*, we identified expansions of the caspase gene family, C-type lectin-like proteins, and inhibitor of apoptosis proteins that can bind to and inhibit caspases and other proteins involved in apoptosis. In particular, we found a high abundance of caspases in *C. augens* (35 proteins), whereas in the 12 other arthropods under consideration, the counts range from four in *D. plexippus* to 18 in *D. pulex* (supplementary table 11, Supplementary Material online). Whether or not the expansion of gene families linked to apoptosis and regeneration is indeed related to the high regenerative potential of *C. augens* needs further experimental evaluations. Transcriptomic and proteomic studies in tissues under regeneration might help to explain these observed genomic patterns.

### Endogenous Viral Elements in the Genome of *C. augens*

**Fig. 5.**
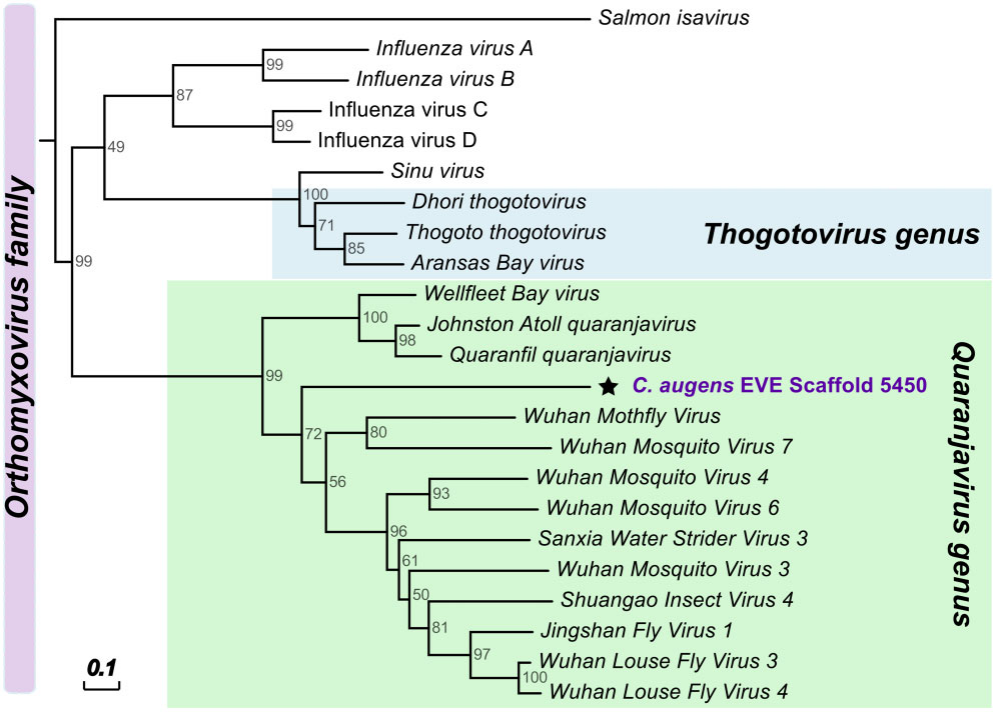
—Phylogenetic relationships of the putative endogenous viral elements in *Campodea augens* genome related to -ssRNA viruses of the Orthomyxoviridae family. All endogenous viral elements found in *C. augens* correspond to orthomyxoviral Polymerase Basic protein 1 (PB1) (Pfam Id PF00602; “Flu_PB1”) (see also supplementary figs. 12 and 13, Supplementary Material online). Neighbor-joining trees was constructed using amino acid sequences of EVEs and BP1 proteins of representatives of the Orthomyxoviridae family. Support for trees was evaluated using 1,000 pseudo replicates. Node values correspond to the bootstrap support. Scale bar indicate amino acid substitutions per site. Green box highlights viruses of the Quaranjavirus genus, whereas light blue box indicates the Thogotovirus genus.

We found seven fragments of nonretroviral integrated RNA viruses (NIRVs) in the genome of *C. augens* that resemble viral sequences of the genus Quaranjavirus, with the highest similarity to Wuhan Louse Fly Virus 3, Wuhan Mosquito Virus 3 and 5, Shuangao Insect Virus 4, and Wellfleet Bay Virus (supplementary table 12, Supplementary Material online). Quaranjaviruses belong to Orthomyxoviridae, a family of segmented negative single-stranded RNA viruses (-ssRNA) that also include the influenza viruses (Presti et al. 2009). All quaranjavirus-like insertions found in *C. augens* encode the PB1 protein (polymerase basic protein 1) (supplementary table 12, Supplementary Material online), a subunit of the RNA-dependent RNA polymerase (RdRp), which in Orthomyxoviridae is typically a heterotrimeric complex (formed by the PA, PB1, and PB2 proteins) (Stubbs and te Velthuis 2014), contrary to RdRps from other RNA viruses (e.g., double-stranded and positive-stranded RNA viruses) that are typically monomeric (Wolf et al. 2018). The corresponding open reading frames of PB1 proteins are incomplete, though, and contain stop codons and/or frameshift mutations (supplementary Fasta, Supplementary Material online), suggesting that these elements are not functional. As indicated by the phylogenetic analyses in figure 5 and supplementary figures 13 and 14, Supplementary Material online, the putative viral insertions are more closely related to a cluster of quaranjaviruses that have been identified in arthropod samples through metagenomic RNA sequencing (Li et al. 2015) than to other quaranjaviruses (e.g., Johnston Atoll quaranjavirus and Quaranfil quaranjavirus) (supplementary table 12, Supplementary Material online). Only one case of a quaranjavirus-like insertion has been previously described in the genome of the black-legged tick (Katzourakis and Gifford 2010). However, this sequence is more closely related to that of Johnston Atoll quaranjavirus and encodes a different viral protein (supplementary fig. 14, Supplementary Material online). The presence of orthomyxovirus-like NIRVs in *C. augens* is of particular interest because Orthomyxoviridae include the Influenza viruses. Recent studies have suggested a central role of arthropods in the origin and evolution of viral species (Li et al. 2015; Dudas and Obbard 2015; Shi et al. 2016), and NIRVs are instrumental for studying virus–host interactions (Aswad and Katzourakis 2016; Olson and Bonizzoni 2017) and to gain insights on viral long-term evolution.

## Conclusions

We sequenced and annotated the 1.2-Gb genome of *C. augens*, a blind and ancestrally wingless hexapod belonging to the systematic order Diplura. Gene structure analysis highlighted a genome-wide trend of remarkably long introns that span 27% of the assembly. We identified a paucity of photoreceptor genes mirroring at the genomic level the secondary loss of an ancestral external photoreceptor organ, and by contrast the presence of the largest ionotropic receptor gene repertoire in the animal kingdom, which account for ∼8% of the total *C. augens* gene set. We further detected expansions of gene families related to detoxification and carbohydrate metabolism, which might reflect adaptations in *C. augens*’ foraging behavior. To date, most of the existing and recent experimental data on diplurans derive from *C. augens* and *C. aquilonaris*, for example, studies on brain anatomy and circulatory organs (Gereben-Krenn and Pass 1999; Böhm et al. 2012). We thus think *C. augens* and *C. aquilonaris* represent good candidates as model species for diplurans. The availability of their genomes can enable detailed molecular studies and unlock the potential of linking diplurans’ peculiar morphological/behavioral features to genetic evidence. The *C. augens* genome opens up novel opportunities to study both the underexplored biology of diplurans as well as the origin and evolution of gene families in insects.

## Data Availability

This Whole Genome Shotgun project has been deposited at DDBJ/ENA/GenBank under the accession VUNT00000000. The version described in this paper is version VUNT01000000. Raw sequencing data have been deposited in the NCBI Sequence Read Archive (SRA) under the accessions SRX3424039–SRX3424046. The annotated mitochondrial genome sequence was deposited in GenBank under the accession number MN481418. The genome and mitochondrial assemblies, gene set, and additional data such as the short scaffolds (<1 kb), the curated chemoreceptor genes, and the supermatrix used for the species phylogeny in figure 2 can also be obtained from http://cegg.unige.ch/campodea_augens; last accessed November 29, 2019. Credentials for accessing the Apollo browser are provided upon request.

## Supplementary Material

Supplementary data are available at *Genome Biology and Evolution* online.

## Supplementary Material

evz260_Supplementary_Data

## Notes

**Data deposition:** This project has been deposited at NBCI under the BioProject accession PRJNA416902.
