# Supplementary material for: The Genome of the Blind Soil-Dwelling and Ancestrally Wingless Dipluran Campodea augens: A Key Reference Hexapod for Studying the Emergence of Insect Innovations
Source: Genome Biol Evol. 2019 Dec 3;12(1):3534–49. doi: 10.1093/gbe/evz260 (PMC6938034; doi:10.1093/gbe/evz260)
Supplement: evz260_Supplementary_Data [file evz260_supplementary_data.zip › Supplementary_file.pdf]

Supplementary Materials for

The genome of the blind soil-dwelling and ancestrally wingless dipluran *Campodea augens*, a key reference hexapod for studying the emergence of insect innovations

Mosè Manni[1,*], Felipe A. Simao[1], Hugh M. Robertson[2], Marco A. Gabaglio[1], Robert M. Waterhouse[3], Bernhard Misof[4], Oliver Niehuis[5], Nikolaus U. Szucsich[6], Evgeny M. Zdobnov[1,*]

[1]Department of Genetic Medicine and Development, University of Geneva Medical School, and Swiss Institute of Bioinformatics, Geneva, Switzerland.
[2]Department of Entomology, University of Illinois at Urbana-Champaign, Urbana, IL, USA.
[3]Department of Ecology and Evolution, University of Lausanne, and Swiss Institute of Bioinformatics, Lausanne, Switzerland.
[4]Center for Molecular Biodiversity Research, Zoological Research Museum Alexander Koenig, Bonn, Germany.
[5]Department of Evolutionary Biology and Ecology, Albert Ludwig University, Institute of Biology I (Zoology), Freiburg, Germany.
[6]Natural History Museum Vienna, 3rd Zoological Dept., Vienna, Austria.

* Corresponding author:

- Evgeny M. Zdobnov, Department of Genetic Medicine and Development, University of Geneva Medical School, and Swiss Institute of Bioinformatics, Geneva, Switzerland, telephone number: +41 (0)22 379 59 73, fax number: +41 (0)22 379 57 06, email: evgeny.zdobnov@unige.ch.
- Mosè Manni, Department of Genetic Medicine and Development, University of Geneva Medical School, and Swiss Institute of Bioinformatics, Geneva, Switzerland, telephone number: + 41 (0)22 379 5432, email: mose.manni@unige.ch.

Contents

SUPPLEMENTARY NOTES....................4
Contamination....................4
Mitochondrial genome....................4
Phylogenomic analysis....................4
Chemoreceptors....................6
Expansion of gene families related to sugar metabolism and transport....................7
SUPPLEMENTARY TABLES....................8
Table S1: Summary of *C. augens* sequencing libraries....................9
Table S2: Statistics of *C. augens* genome compared to the four available ancestrally wingless hexapod genomes, and other insects with large genomes....................10
Table S3: Repeat content of *C. augens* genome....................11
Table S4: Gene composition of *C. augens* compared to the other ancestrally wingless hexapods....................14
Table S5: BUSCO analysis of *C. augens* assembly and gene set along with busco scores for the other available ancestrally wingless hexapods. BUSCO v3 was run using arthropoda_odb9 dataset....................15
Table S6: Bacterial hits in *C. augens* genome....................16
Table S7: Characteristics of *C. augens* mitogenome....................18
Table S8: Significant gene family contractions and expansions in *C. augens*....................20
Table S9: Gene families in *C. augens* that are significantly larger than the mean counts in insects....................23
Table S10: Counts of Pfam domains associated with DNA photolyase in *C. augens* and 12 other arthropods....................29
Table S11: Counts of Pfam domains associated with the caspase domain in *C. augens* and 12 other arthropods....................30
Table S12: Blast hit and Pfam domains of *C. augens* EVEs....................31
Table S13: Accession number of PB1 proteins from orthomyxoviruses used in the phylogenetic analysis of *C. augens* EVEs....................32
Table S14: Source of genomic data used in the analyses....................33
SUPPLEMENTARY FIGURES....................35
Figure S1a: High-resolution picture of *C. augens*....................36
Figure S1b: High-resolution picture of *C. augens*....................37
Figure S2: Assembly and annotation workflow used for *C. augens*....................38
Figure S3: Cumulative distributions of scaffold lengths in *C. augens* and other arthropods....................39
Figure S4: Plots of BUSCO scores for *C. augens* and the other ancestrally wingless hexapods using the arthropoda_v9 dataset....................40
Figure S5: Hox genes cluster arrangement for *Campodea augens, Daphnia magna, Folsomia candida* and *Drosophila melanogaster*....................41
Figure S6: Gene, intron and exon length distributions in *C. augens* and other ancestrally wingless hexapods....................42
Figure S7: Graphical representation of the mitochondrial genomes of *C. augens* and *C. fragilis*....................43
Figure S8: Ultrametric species phylogeny displaying expansions/contractions of gene families as estimated using CAFE....................44
Figure S9: Heat map of OrthoDB clusters that are significantly larger in *C. augens* compared to the mean counts from insect species....................45

Figure S10: Phylogenetic tree of the IR family....46
Figure S11: Histogram of the numbers of pseudogenes with 1-8 pseudogenizing mutations....47
Figure S12: Genomic regions encoding a cluster of 5 CYP450 with 4 GST genes (Scaffold_1992), and a cluster of 6 UGTs (Scaffold_1829)....48
Figure S13: MSA of a putative endogenous viral elements in *C. augens* genome (C. augens_EVE_5450) and PB1 sequences of Orthomyxoviridae....49
Figure S14: Phylogenetic relationships of the EVEs found in *C. augens* related to -ssRNA viruses of the Orthomyxoviridae family....50
REFERENCES....51

SUPPLEMENTARY NOTES

Contamination

By scanning raw reads for bacterial contamination, we detected a small number of reads with high nucleotide similarity to fragments of the *Wolbachia* genome. *Wolbachia* endosymbionts commonly infect arthropods and can occasionally transfer parts of their genome to the nuclear genome of their arthropod host (Robinson et al. 2013). The low abundance of reads possibly originating from a *Wolbachia* genome suggests that the samples used for sequencing the genome of *C. augens* were likely not infected by the endosymbiont. We consider it more likely that the *Wolbachia*-like reads originated by historical Lateral Gene Transfer (LGT) events. In total, we found on 24 scaffolds in the *C. augens* assembly 29 short nucleotide sequence sections of putative bacterial origin and likely incorporated into the genomes by LGT (Supplementary Table 6).

Mitochondrial genome

The mitochondrial genome (mitogenome) of *C. augens* was assembled separately from the nuclear genome using the de novo assembler NOVOPlasty v2.6.7, resulting in an assembly of 15,735 bp in length, a size comparable to that of the mitogenome of *Campodea fragilis* (14,965 bp) (Podsiadlowski et al. 2006) (Supplementary Fig. 7).

The annotation of the *C. augens* mitogenome was performed using the web version of the metazoan mt genome annotator MITOS (Bernt et al. 2013), using the invertebrate genetic code and the RefSeq 63 Metazoa as reference database. The *C. augens* mitogenome encodes for 13 protein-coding genes, 22 tRNAs, and two rRNAs. The order of genes is identical to *Campodea fragilis* (Supplementary Table 7, Supplementary Fig. 7). The AT-rich control region (around 1 Kbp in size) is located between the *trnL* and *rrnS* genes.

Phylogenomic analysis

A phylogenomic analysis was conducted using *C. augens* and 13 other arthropod species of interest including a) insect species: *Drosophila melanogaster* (fruit fly), *Danaus plexippus* (monarch butterfly), *Tribolium castaneum* (red flour beetle), *Apis mellifera* (honey bee), *Pediculus humanus* (body louse), *Acyrthosiphon pisum* (pea aphid), *Calopteryx splendens* (banded demoiselle); b) representatives of Collembola, Diplura and Protura: *Folsomia candida* (springtail), *Orchesella cincta* (springtail), *Catajapyx aquilonaris* (northern forcepstail), *Acerentomon sp.* (coneheads); and c) two non-hexapod species: *Daphnia pulex* (water flea) and *Strigamia marítima* (centipede).

The order Prutura (coneheads) still lack a sequenced genome, but a representative of this taxon is crucial in the context of our phylogenetic analysis to resolve the relationships among ancestrally wingless hexapod. Therefore we re-assembled the transcriptome of *Acerentomon sp.* from raw reads deposited at SRA (SRR921562), produced in the context of 1KITE project, and used it as the source of phylogenomic markers. Assembly of the transcriptome was performed using Trinity (Grabherr et al. 2011) with default parameters. For extracting single-copy genes shared among the 13 species of interest mentioned above we used BUSCO following the pipeline described in (Waterhouse et al. 2018), which allows to extract single-copy genes using as input gene sets, genomes or transcriptomes (scripts available at https://gitlab.com/ezlab/busco_usecases/tree/master/phylogenomics). For building the phylogenetic tree, we employed 358 single-copy genes, out of the ca. 1000 universal single-copy genes expected to be found in arthropoda according to BUSCO arthropoda dataset (Waterhouse et al. 2018; Kriventseva et al. 2019), that were present in all the 14 species under consideration, thus minimizing missing data in the matrix. The corresponding OrthoDB IDs of these 358 genes can be found in the text file of partitions "Species_phylogeny_partitions.txt" at http://cegg.unige.ch/campodea_augens. The corresponding description can be retrieve from ODB9 website (https://www.orthodb.org/v9/index.html) by using the ID as query. The amino acid sequences of the 358 single-copy genes shared among the 13 species were then aligned individually, with MAFFT (Katoh & Standley 2013) using the L- INS-i algorithm. Each protein multiple sequence alignment (MSA) was visually inspected to detect presence of misalignments. Each MSA were subsequently trimmed with Trimal v1.2.59 for automatic trimming using the "-auto" option. MSAs were then concatenated in a supermatrix 145,145 aa long. The phylogenetic analysis was performed using a partitioned model-based tree reconstruction, considering the selected single-copy genes as evolutionary units. We

conducted a meta-partition analysis using the protTest software version 3.4.2 (Darriba et al. 2011). protTest was run on each individual trimmed MSA, and the best model according to AICc was assigned to each MSA. The MSA and partition file are available at http://cegg.unige.ch/campodea_augens. To infer the phylogenetic tree based on a Maximum Likelihood (ML) approach we used the PTHREADS version of RAxML v8 (Stamatakis 2014). The partition information derived with protest was provided to RAxML using the “-q” option. We estimated 100 bootstrap replicates and drawn bootstrap values on the best ML tree using the standard RAxML options. Tree was rooted by selecting S*trigamia maritima* as the outgroup species, and it was annotated using the R package ggtree (Yu et al. 2017). Alternatively, we also analyzed the whole matrix using various mixture models implemented in IQ-TREE v1.6.10 (Nguyen et al. 2015). Support values were estimated by ultrafast bootstrapping for 1000 replicates. The results were congruent with the topology obtained using partitions in RAxML, placing Diplura as a sister clade to Insecta. The trees obtained with IQ-TREE using different mixture models are reported below:

EX_EHO model:
(((ACERE:0.3722466053,(FCAND:0.1932572727,OCINC:0.2238088850)100:0.2884263193)98:0.0367511722,((((AMELL:0.2573580956,
((DMELA:0.3680389924,DPLEX:0.3146272518)100:0.0517523676,TCAST:0.2464391402)100:0.0390115020)100:0.0300424702,
(APISU:0.4196732606,PHUMA:0.2834345904)100:0.0378272404)100:0.0313712042,CSPLE:0.2512345655)100:0.0629332830,
(CAQUI:0.2327130107,CAUGE:0.3176562358)100:0.0728476348)86:0.0196511931)100:0.0409085274,DPULE:0.3468198516,SMARI:0.3432093
826);

EX2 model:
(((ACERE:0.3653026123,(FCAND:0.1938355157,OCINC:0.2221617439)100:0.2801976402)99:0.0397104878,((((AMELL:0.2547959289,
((DMELA:0.3606424543,DPLEX:0.3093933852)100:0.0541921806,TCAST:0.2435941194)100:0.0410729731)100:0.0320518128,
(APISU:0.4105711164,PHUMA:0.2791013121)100:0.0405677499)100:0.0335293535,CSPLE:0.2501829340)100:0.0644800555,
(CAQUI:0.2315965170,CAUGE:0.3130919435)100:0.0734698875)91:0.0221596281)100:0.0427428037,DPULE:0.3407857788,SMARI:0.3374211
934);

EX3 model:
(ACERE:0.3688243196,(((((AMELL:0.2545129145,
((DMELA:0.3648701223,DPLEX:0.3122653738)100:0.0528636633,TCAST:0.2452033362)100:0.0398988541)100:0.0308996506,
(APISU:0.4142856836,PHUMA:0.2812713489)100:0.0386355939)100:0.0321504263,CSPLE:0.2520255112)100:0.0638158811,
(CAQUI:0.2335132755,CAUGE:0.3146898327)100:0.0733888059)85:0.0205965418,
(DPULE:0.3445738398,SMARI:0.3396821478)100:0.0415661780)100:0.0377115529,
(FCAND:0.1925376333,OCINC:0.2228080143)100:0.2858256698);

EHO model:
(((ACERE:0.3425637704,(FCAND:0.1868623586,OCINC:0.2116748237)100:0.2562105105)100:0.0418614319,((((AMELL:0.2436079148,
((DMELA:0.3374428965,DPLEX:0.2904640996)100:0.0543869361,TCAST:0.2311603875)100:0.0404896155)100:0.0322090250,
(APISU:0.3831956100,PHUMA:0.2625109490)100:0.0425219333)100:0.0330212671,CSPLE:0.2371375195)100:0.0604887373,
(CAQUI:0.2194560961,CAUGE:0.2942413438)100:0.0709061967)87:0.0232039777)100:0.0440228678,DPULE:0.3187625410,SMARI:0.3170877
377);

Chemoreceptors

The GR and IR families were manually annotated (see supplementary file Curated_chemosensory_genes.txt). This effort was complicated, especially in the case of the IRs, by three factors. First, the genome assembly has many single-N gaps, which mark the locations of artifactual duplications and more rarely deletions. As these sometimes affect parts of genes, and occasionally entire genes, care was taken not to include such

identical adjacent direct repeat fragments of genes. If such a duplication had occurred within a gene, the genomic sequence was repaired to fix the model, however if a gene terminus was missing due to a single N the assembly was not repaired and the model treated as incomplete. Second, pseudogenes were included if they encoded at least 50% of the length of a related intact IR (these IRs vary in length by two-fold from 445 to 897 amino acids, so no single length criterion could be employed). In most cases pseudogenes result from one or more stop codons or short frameshifting indels, and occasionally loss of either terminus or large internal deletions, however there are some with so many pseudogenizing mutations that they were too difficult to reconstruct and include in the analysis, despite representing more than half length of a gene (generally those with more than seven pseudogenizing mutations). Third, there are innumerable gene fragments encoding less than half of a related intact IR, and most of these are assumed to be pseudogenic fragments and ignored. Each set of closely related IRs was iteratively searched against the genome using TBLASTN searches to find all close relatives in an expanded lineage, and divergent relatives identified in the same scaffolds were sequestered for later searching as a way to discover the entire family (commonly around 20% amino acid identity for only the central relatively-conserved region of the protein). These divergent relatives were then used as queries to explore divergent lineages towards the end of the effort. Extensive searches with each of these divergent lineages eventually found only already annotated genes, suggesting that all IR genes in this genome have been recovered.

As described in the main text, the GR family is modestly-sized with 41 genes, seven of them pseudogenes. The family composition is relatively straightforward with 13 relatives of the sugar receptor subfamily in insects, no relatives of the carbon dioxide and fructose receptors commonly conserved in insects, and 28 otherwise divergent members.

In contrast, the IR family is enormously expanded with 2431 genes and pseudogenes named and included in the analysis. Conserved orthologs were found for the co-receptors Ir8a, 25a, and 76b (named for their *D. melanogaster* orthologs), however Ir25a has been duplicated at least four times, with additional gene fragments suggesting there is a fifth copy. These 4 or 5 Ir25a orthologs might be involved in dimerization with the many divergent IRs found.

The numbers of obvious pseudogenizing mutations (stop codons, frameshifts, and large deletions) were noted for each pseudogene. The histogram in Figure S11 reveals that the majority have single mutations. The caveat noted above applies to the right hand of this histogram, as some reasonably long pseudogenes with

too many mutations were too hard to reconstruct and hence excluded from the set, but the preponderance of pseudogenes with single mutations versus those with 2-5 mutations is real as the latter were consistently reconstructed.

Expansion of gene families related to sugar metabolism and transport

In relation to the foraging ecology of *C. augens*, we also found a number of expanded gene families associated with carbohydrate metabolism and transport. This may reflect adaptations to the carbohydrate-rich diet of *C. augens*. In particular, we identified substantial changes in the gene copy numbers of trehalose receptors, glucose-methanol-choline (GMC) oxidoreductases, glycosyltransferases, histidine phosphatases, and glycosyl hydrolases (Supplementary Tables 8 and 9). Catalytic functions of histidine phosphatases include phytase activity to hydrolyse phytic acid, present in many plants tissues. Glycosyl hydrolases catalyze the hydrolysis of glycosidic bonds in complex sugars and they are involved in the degradation of cellulose, hemicellulose, and starch. Genes involved in sugar transport, such as the major facilitator superfamily (MFS), sodium sulfate symporters, and the EamA-like transporter family, were also found to be expanded (Supplementary Tables 8 and 9).

SUPPLEMENTARY TABLES

Table S1: Summary of *C. augens* sequencing libraries.

Library	Library type	Insert size	Read length (bp)	Number of reads (million)	Sequencing output (Gb)	Genome coverage (X)
350_L1	paired-end	350 bp	100	277,91	27,79	23,16
350_L6	paired-end	350 bp	100	358,09	35,81	29,84
550_L2	paired-end	550 bp	100	270,31	27,03	22,53
550_L7	paired-end	550 bp	100	298,62	29,86	24,89
m3	mate pair	3 kb	100	277,62	27,76	23,13
m6	mate pair	6 kb	100	230,00	23,00	19,17
m9	mate pair	9 kb	100	218,07	21,81	18,17
m12	mate pair	12 kb	100	207,42	20,74	17,29
			Total	2138,03	213,80	178,17

Table S2: Statistics of *C. augens* genome compared to the four available ancestrally wingless hexapod genomes, and other insects with large genomes.

	Non-insect hexapods					Insects				
Metric	*Campodea augens*	*Catajapyx aquilonaris*	*Folsomia candida*	*Orchesella cincta*	*Holacanthella duospinosa*	*Calopteryx splendens*	*Blatella germanica*	*Locusta migratoria*	*Medauroidea extradendata*	*Clitarchus hookeri*
Assembly size (Mb)	1130	312	221	286	328	1630	2040	5760	2600	3800
No. of scaffolds	18,765	132,454	162	9,402	62,430	8,896	24,818	1,397,492	135,691	785,781
No. of contigs	70,883	151,491	228	9,641	70,136	645,677	317,827	1,397,492	167,455	906,073
Scaffold L/N50 (Kb)	1,367/235	2,531/31	8/6520	925/66	242/310	1,013/422	576/1050	174,483/10	17,103/43	2,981/316
Contig L/N50 (Kb)	8,725/33	6,775/12	12/4890	936/64	985/80	83,275/4	39,325/12	174,483/10	26,796/28	21,033/46
Max scaffold length (Mb)	2.19 Mb	0.42	28.53	0.81	2.81	2.78	7.47	0.11	0.43	4.95
Max contig length (Kb)	390	142	20230	807	1270	63	1930	106	250	626
No. of scaffolds > 50 Kb	5,264	1,290	83	1,288	775	5,525	2,997	877	13,531	9,687
Assembly in scaffolds > 50 Kb (%)	90	35	99	57	72	94	93	1	44	76
GC	0.33	0.43	0.37	0.37	0.33	0.39	0.34	0.41	0.37	0.39
Gap (%)	8.4	0.3	0.1	0.0	2.5	18.7	16.0	0.0	0.3	3.1

Table S3: Repeat content of *C. augens* genome.

Type	No. elements	Length (bp)	Percentage
**DNA transposons**	338,259	89,947,607	7.980
hAT-Ac	105,066	27,773,842	2.464
hAT-Tip100	70,523	18,910,018	1.678
hAT-Blackjack	39,085	11,083,906	0.983
Crypton-I	12,082	4,144,513	0.368
RC	17,021	3,827,203	0.340
TcMar-Tc4	9,109	2,352,621	0.209
hAT	7,003	2,295,567	0.204
hAT-Charlie	8,210	2,229,463	0.198
Sola-2	6,920	2,054,882	0.182
TcMar-Mariner	5,163	2,025,793	0.180
hAT-Tag1	4,661	1,406,650	0.125
Sola-1	7,536	1,269,310	0.113
PIF-Harbinger	5,513	1,225,180	0.109
hAT-hATx	2,798	997,184	0.088
hAT-hAT5	3,947	839,482	0.074
CMC-Chapaev-3	1,655	685,463	0.061
MULE-MuDR	2,586	675,591	0.060
TcMar-Tc1	2,785	663,887	0.059
CMC-EnSpm	4,390	607,351	0.054
PiggyBac	1,480	605,286	0.054
Merlin	1,977	549,482	0.049
MuLE-MuDR	1,809	509,286	0.045
CMC-Transib	5,909	458,536	0.041
Crypton	1,900	442,963	0.039
hAT-hAT19	1,298	437,367	0.039
Crypton-V	1,427	399,974	0.035
IS3EU	1,379	326,398	0.029
Academ-1	1,502	316,671	0.028
Maverick	1,150	316,523	0.028
Sola	571	205,488	0.018
TcMar-Tc2	1,204	151,704	0.013
Kolobok-T2	375	125,298	0.011

hAT-hATm	106	23,23	0.002
hAT-Pegasus	45	4,211	0.000
hAT-hobo	36	3,702	0.000
MULE-NOF	23	2,079	0.000
Zator	12	1,36	0.000
Kolobok-Hydra	2	104	0.000
TcMar-Pogo	1	39	0.000
**Retroelements**	1,588,521	380,226,633	33.733
**LINEs**	96,449	25,730,249	2.283
Penelope	53,537	12,204,040	1.083
L3	14,604	4,263,651	0.378
CR2	10,535	3,625,019	0.322
RTE-X	3,636	1,669,624	0.148
Jockey	3,906	1,172,241	0.104
L1-Tx2	4,752	963,984	0.086
Dong-R5	1,041	559,161	0.050
L2	741	468,809	0.042
RTE-BovB	1,381	356,411	0.032
CR1-Zenon	387	143,446	0.013
I-Jockey	1,169	142,842	0.013
I-Nimb	204	85,242	0.008
CRE-II	210	55,67	0.005
R2	255	14,491	0.001
R1-LOA	39	2,209	0.000
R3	35	1,826	0.000
R2-NeSL	17	1,583	0.000
**SINEs**	25,772	4,257,031	0.378
tRNA	11,654	1,680,388	0.149
tRNA-Deu-RTE	10,098	1,678,969	0.149
MIR	1,738	505,956	0.045
tRNA-L3	1,419	263,956	0.023
tRNA-V	863	127,762	0.011
**LTR elements**	1,466,300	350,239,353	31.072
Gypsy	26,969	7,222,488	0.641
Copia	20,279	3,592,811	0.319
Pao	2,101	932,997	0.083

Ngaro	1,378	569,249	0.051
ERVL	2,219	485,597	0.043
ERV1	1,424	350,196	0.031
**Unknown**	2002392	376811916	33.43
**Low complexity**	86	27,471	0.002
**Satellites**	2,644	631,963	0.056
**Simple repeats**	28,426	4,938,721	0.438
**Total repeats**		475,772,395	45.734

Table S4: Gene composition of *C. augens* compared to the other ancestrally wingless hexapods.

	Diplura		Collembola		
Metric	*Campodea augens*	*Catajapyx aquilonaris*	*Folsomia candida*	*Orchesella cincta*	*Holacanthella duospinosa*
Assembly size (Mb)	1130	312	221	286	327
No. of protein-coding genes	23,992	10,901	22,100	20,247	9,895
Total gene length (Mb)	338	39	94	61	57
Genome covered by genes (%)	30.0	12.6	42.3	21.1	17.3
Mean gene length (bp)	14,081	3,609	4,240	2,990	5,726
Median gene length (bp)	9,728	2,529	2,240	2,043	3,784
Total size of exons (Mb) / (%)	31.9 / (2.83)	15.5 / (4.96)	53.1 / (23.94)	36.2 / (12.63)	22.7 / (6.93)
Mean exon length (bp)	227	250	256	306	285
Total size of introns (Mb) / (%)	306.2 / (27,17)	24.0 / (7,68)	81.5 / (36,77)	24.5 / (8,55)	34.1 / (10.41)
Mean intron length (bp)	2631	468	449	250	489
Median intron length (bp)	1636	128	88	90	107
Mean exons per gene	6	6	8	6	8
Mean introns per gene	5	5	7	5	7

*Intron and exon sizes were calculated based on the GFF files of the corresponding genomes (see Table SX for data sources).

Table S5: BUSCO analysis of *C. augens* assembly and gene set along with busco scores for the other available ancestrally wingless hexapods. BUSCO v3 was run using arthropoda_odb9 dataset.

Species	Complete + Fragmented	Complete	Complete and single-copy	Complete and duplicated	Fragmented	Missing
Campodea augens	97.6	91.8	89.8	2.0	5.8	2.4
	97.0	90.4	86.6	3.8	6.6	3.0
Catajapyx aquilonaris	99.3	97.0	94.9	2.1	2.3	0.7
	96.9	92.3	90.2	2.1	4.6	3.1
Folsomia candida	97.2	96.1	94.7	1.4	1.1	2.8
	98.6	97.8	72.0	2.2	0.8	1.4
Orchesella cincta	96.6	95.4	87.4	8.0	1.2	3.4
	92.2	88.5	81.6	6.9	3.7	7.8
Holacanthella duospinosa	83.9	80.4	73.6	6.8	3.5	16.1
	83.3	76.7	69.4	7.3	6.6	16.7

*Grey lines refers to busco scores for gene sets, blank lines to scores for genomes.

Table S6: Bacterial hits in *C. augens* genome.

	Scaffold	HGT candidate	Start	Stop	Strand	Best hit gene ID	Clade	Species	Description
1	Scaffold_283	CAUGE_02727	69101	71989	-	gi|1127377281|gb|APR98680.1|	a-proteobacteria	Wolbachia endosymbiont of Folsomia candida	hypothetical protein ASM33_05545 [Wolbachia endosymbiont of Folsomia candida]
2	Scaffold_283	CAUGE_02728	81704	85936	-	gi|1127377281|gb|APR98680.1|	a-proteobacteria	Wolbachia endosymbiont of Folsomia candida	hypothetical protein ASM33_05545 [Wolbachia endosymbiont of Folsomia candida]
3	Scaffold_364	CAUGE_03437	144611	182926	-	gi|736445120|ref|WP_034466997.1|	a-proteobacteria	Afipia sp. P52-10	CoA transferase subunit B [Afipia sp. P52-10]
4	Scaffold_433	CAUGE_03961	57354	79310	-	gi|505401443|ref|WP_015588545.1|	a-proteobacteria	Wolbachia endosymbiont of Drosophila simulans	Ankyrin repeat domain protein [Wolbachia endosymbiont of Drosophila simulans]
5	Scaffold_501	CAUGE_04532	228425	236587	-	gi|1267475813|ref|WP_098291706.1|	firmicutes	Bacillus thuringiensis	hypothetical protein [Bacillus thuringiensis]
6	Scaffold_516	CAUGE_04651	609623	630080	+	gi|662720153|gb|AIE61363.1|	firmicutes	Bacillus methanolicus MGA3	hypothetical protein BMMGA3_15030 [Bacillus methanolicus MGA3]
7	Scaffold_550	CAUGE_04872	44396	59277	+	gi|1169884537|ref|WP_080518662.1|	b-proteobacteria	Burkholderia mallei	hypothetical protein [Burkholderia mallei]
8	Scaffold_1016	CAUGE_07765	285612	315167	+	gi|1131919362|emb|SIT08514.1|	a-proteobacteria	Insolitispirillum peregrinum	Ca2+-binding protein, RTX toxin-related [Insolitispirillum peregrinum]
9	Scaffold_1250	CAUGE_08970	230	5447	+	gi|503535025|ref|WP_013769101.1|	CFB group bacteria	Haliscomenobacter hydrossis	hypothetical protein [Haliscomenobacter hydrossis]
10	Scaffold_1281	CAUGE_09116	101679	102350	+	gi|1154081569|ref|WP_078460720.1|	g-proteobacteria	Solemya velum gill symbiont	hypothetical protein [Solemya velum gill symbiont]
11	Scaffold_1309	CAUGE_09231	342507	346766	+	gi|1127377281|gb|APR98680.1|	a-proteobacteria	Wolbachia endosymbiont of Folsomia candida	hypothetical protein ASM33_05545 [Wolbachia endosymbiont of Folsomia candida]
12	Scaffold_1309	CAUGE_09232	371494	373902	+	gi|1244309946|gb|PBQ26533.1|	a-proteobacteria	Wolbachia pipientis wAus	hypothetical protein BTO27_04595, partial [Wolbachia pipientis wAus]
13	Scaffold_1309	CAUGE_09233	401769	405515	+	gi|1031777381|ref|WP_064125268.1|	a-proteobacteria	Wolbachia endosymbiont of Dactylopius coccus	hypothetical protein [Wolbachia endosymbiont of Dactylopius coccus]
14	Scaffold_1587	CAUGE_10534	27565	28146	+	gi|1200292621|gb|OUU14650.1|	a-proteobacteria	Alphaproteobacteria bacterium TMED37	hypothetical protein CBB97_24755 [Candidatus Endolissoclinum sp. TMED37]

15	Scaffold_1587	CAUGE_10535	31594	32148	+	gi|1200292621|gb|OUU14650.1|	a-proteobacteria	Alphaproteobacteria bacterium TMED37	hypothetical protein CBB97_24755 [Candidatus Endolissoclinum sp. TMED37]
16	Scaffold_1587	CAUGE_10536	34910	35467	+	gi|1200292621|gb|OUU14650.1|	a-proteobacteria	Alphaproteobacteria bacterium TMED37	hypothetical protein CBB97_24755 [Candidatus Endolissoclinum sp. TMED37]
17	Scaffold_1633	CAUGE_10756	299421	307144	+	gi|981458280|ref|WP_059667283.1|	b-proteobacteria	Burkholderia ubonensis	hypothetical protein [Burkholderia ubonensis]
18	Scaffold_3169	CAUGE_15978	107069	144500	-	gi|1127377281|gb|APR98680.1|	a-proteobacteria	Wolbachia endosymbiont of Folsomia candida	hypothetical protein ASM33_05545 [Wolbachia endosymbiont of Folsomia candida]
19	Scaffold_3228	CAUGE_16137	15620	19066	+	gi|1180269025|ref|WP_083787830.1|	a-proteobacteria	Rickettsia endosymbiont of Ixodes scapularis	hypothetical protein [Rickettsia endosymbiont of Ixodes scapularis]
20	Scaffold_3582	CAUGE_16967	327	5679	+	gi|494597110|ref|WP_007355367.1|	cyanobacteria	Oscillatoria sp. PCC 6506;Kamptonema	MULTISPECIES: hypothetical protein [Kamptonema]
21	Scaffold_3959	CAUGE_17788	57789	60155	+	gi|1127377281|gb|APR98680.1|	a-proteobacteria	Wolbachia endosymbiont of Folsomia candida	hypothetical protein ASM33_05545 [Wolbachia endosymbiont of Folsomia candida]
22	Scaffold_4330	CAUGE_18555	1289	11179	+	gi|501450016|ref|WP_012473465.1|	CFB group bacteria	Candidatus Amoebophilus asiaticus	hypothetical protein [Candidatus Amoebophilus asiaticus]
23	Scaffold_4450	CAUGE_18804	41770	42873	+	gi|1234008076|ref|WP_094649383.1|	a-proteobacteria	Rickettsia endosymbiont of Culicoides newsteadi	translational GTPase TypA [Rickettsia endosymbiont of Culicoides newsteadi]
24	Scaffold_6069	CAUGE_20726	5785	6549	-	gi|1127377281|gb|APR98680.1|	a-proteobacteria	Wolbachia endosymbiont of Folsomia candida	hypothetical protein ASM33_05545 [Wolbachia endosymbiont of Folsomia candida]
25	Scaffold_18317	CAUGE_23038	28848	35599	-	gi|942689125|ref|WP_055393802.1|	CFB group bacteria	Flagellimonas eckloniae	collagen-like protein [Flagellimonas eckloniae]
26	Scaffold_18509	CAUGE_23530	97286	106692	-	gi|1084511163|gb|OGN73343.1|	bacteria	Chlamydiae bacterium RIFCSPLOWO2_12_FULL_49_12	hypothetical protein A3G30_02530 [Chlamydiae bacterium RIFCSPLOWO2_12_FULL_49_12]
27	Scaffold_18672	CAUGE_23831	107631	108875	-	gi|488851699|ref|WP_002764105.1|	spirochetes	Leptospira sp. 200901116	hypothetical protein [Leptospira mayottensis]
28	Scaffold_172	CAUGE_01697	183982	226051	-	gi|857977947|emb|CEO95922.1|	cercozoans	Plasmodiophora brassicae	hypothetical protein PBRA_004612 [Plasmodiophora brassicae]
29	Scaffold_1009	CAUGE_07722	198811	205880	-	gi|914546906|gb|KOB60119.1|	apicomplexans	Plasmodium falciparum HB3	hypothetical protein PFHG_01882 [Plasmodium falciparum HB3]

Table S7: Characteristics of *C. augens* mitogenome.

Name	Start	Stop	Strand	Length (bp)	ovl/nc	Start/Stop Codons	Length in *C. fragilis*	*Length in C. lubbocki*	*Length in D. pulex*
trnI(gat)	1	62	+	62	-3		62	61	64
trnQ(ttg)	60	126	-	67	15		65	64	68
trnM(cat)	142	205	+	64	-12		64	64	64
nad2	194	1213	+	1020	-2	ATT/TAA	1005	1005	988
trnW(tca)	1212	1277	+	66	-1		64	66	66
trnC(gca)	1277	1337	-	61	0		62	53	64
trnY(gta)	1338	1398	-	61	-2		63	62	64
cox1	1397	2935	+	1539	-5	TTG/TAA	1540	1542	1538
trnL2(taa)	2931	2992	+	62	-21		64	63	68
cox2	2972	3656	+	685	15	ATA/T(AA)	679	684	679
trnK(ctt)	3672	3737	+	66	-2		57	68	70
trnD(gtc)	3736	3797	+	62	0		63	61	65
atp8	3798	3953	+	156	-7	ATA/TAA	156	156	162
atp6	3947	4621	+	675	3	ATG/TAA	675	675	674
cox3	4625	5411	+	787	0	ATG/T(AA)	787	787	789
trnG(tcc)	5412	5470	+	59	6		59	60	61
nad3	5477	5824	+	348	-2	ATA/TAG	357	347	353
trnA(tgc)	5823	5890	+	68	0		62	60	66
trnR(tcg)	5891	5941	+	51	-3		52	53	65
trnN(gtt)	5939	5999	+	61	-2		62	61	67
trnS1(gct)	5998	6052	+	55	-1		54	55	65

trnE(ttc)	6052	6114	+	63	-2		64	61	68
trnF(gaa)	6113	6174	-	62	-1		61	60	66
nad5	6174	7880	-	1707	0	ATT/TAG	1707	1710	1708
trnH(gtg)	7881	7942	-	62	-17		64	60	64
nad4	7926	9266	-	1341	-7	ATG/TAA	1338	1326	1321
nad4l	9260	9544	-	285	5	ATG/TAA	285	285	276
trnT(tgt)	9550	9610	+	61	0		60	61	65
trnP(tgg)	9611	9677	-	67	5		67	63	65
nad6	9683	10189	+	507	-19	ATT/TAA	510	524	513
cob	10288	11373	+	1086	-47	ATG/TAG	1143	1140	1134
trnS2(tga)	11327	11379	+	53	14		56	55	69
nad1	11427	12338	-	912	-32	ATA/TAG	924	921	936
trnL1(tag)	12354	12414	-	61	33		63	59	67
rrnL	12419	13460	-	1041			1093	1096	1314
trnV(tac)	13508	13567	-	60	602		62	61	72
rrnS	13575	14258	-	683			722	738	753
Putative Control Region	14648	15673	+	1025			557	620	689

Table S8: Significant gene family contractions and expansions in *C. augens*.

"Family size change" corresponds to number of significantly expanded (+) and contracted (-) gene families (Viterbi p-value < 0.01), numbers in parentheses correspond to family size at the most recent common ancestor and family size in *C. augens*. Domain and domain description correspond to the most abundant domain which occurs in proteins of the cluster. Number of genes is the number of proteins in the cluster, with values in parentheses indicating the number of proteins which contains the corresponding most abundant domain.

Cluster	Family size change	Most abundant domain	Domain description of the most abundant domain	Number of genes
2960	+5 (1 -> 6)	PF00027	Cyclic nucleotide-binding domain	19 (15)
5412	+6 (4 -> 10)	PF02801	Beta-ketoacyl synthase, C-terminal domain	66 (55)
8640	+15 (14 -> 29)	PF00005	ABC transporter	13 (134)
13331	+36 (14 -> 50)	PF00083	Sugar (and other) transporter	13 (122)
17578	+12 (8 -> 20)	PF00732	GMC oxidoreductase	69 (69)
27531	+10 (4 -> 14)	PF06974	Protein of unknown function (DUF1298)	92 (69)
27628	+11 (7 -> 18)	PF00149	Calcineurin-like phosphoesterase	676 (64)
33010	+60 (5 -> 65)	PF00651	BTB/POZ domain	757 (74)
33393	+4 (2 -> 6)	PF04937	Protein of unknown function (DUF 659)	50 (34)
104662	+15 (7 -> 22)	PF00083	Sugar (and other) transporter	76 (75)
39781	+33 (4 -> 37)	PF00060	Ligand-gated ion channel (Ionotropic glutamate receptor)	50 (48)
40422	+11 (14 -> 25)	PF04083	Partial alpha/beta-hydrolase lipase region	227 (173)
42033	+17 (4 -> 21)	PF00135	Carboxylesterase family	52 (52)
44392	+16 (5 -> 21)	PF00501	AMP-binding enzyme	52 (52)
47035	+40 (26 -> 66)	PF00067	Cytochrome P450	357 (355)
47488	+8 (8 -> 16)	PF07690	Major Facilitator Superfamily	109 (94)
49279	+4 (1 -> 5)	PF00083	Sugar (and other) transporter	33 (32)
49470	+16 (7 -> 23)	PF00201	UDP-glucoronosyl and UDP-glucosyl transferase	97 (97)
49474	+5 (2 -> 7)	PF13516	Leucine Rich repeat	40 (4)
49785	+22 (20 -> 42)	PF00067	Cytochrome P450	255 (255)
51218	+8 (6 -> 14)	PF00232	Glycosyl hydrolase family 1	101 (101)
53356	+30 (10 -> 40)	PF00939	Sodium:sulfate symporter transmembrane region	91 (88)
53444	+39 (14 -> 53)	PF00201	UDP-glucoronosyl and UDP-glucosyl transferase	193 (190)
53491	+10 (2 -> 12)	PF14291	Domain of unknown function (DUF4371)	83 (19)
53706	+5 (3 -> 8)	PF00651	BTB/POZ domain	51 (50)
53800	+16 (4 -> 20)	PF00653	Inhibitor of Apoptosis domain	54 (51)

56848	+19 (5 -> 24)	PF00656	Caspase domain	51 (48)
58929	+27 (5 -> 32)	PF08395	7tm Chemosensory receptor	79 (1)
59208	+19 (4 -> 23)	PF02932	Neurotransmitter-gated ion-channel transmembrane region	35 (32)
59400	+7 (4 -> 11)	PF06151	Trehalose receptor	55 (43)
60485	+8 (3 -> 11)	PF00060	Ligand-gated ion channel (Ionotropic glutamate receptor)	45 (18)
63073	+11 (6 -> 17)	PF00089	Trypsin	51 (50)
64020	+14 (6 -> 20)	PF05577	Serine carboxypeptidase S28	66 (66)
64853	+5 (3 -> 8)	PF00665	Integrase core domain	65 (1)
66494	+15 (5 -> 20)	PF00078	Reverse transcriptase (RNA-dependent DNA polymerase)	89 (22)
68788	+23 (8 -> 31)	PF00487	Fatty acid desaturase	112 (106)
75433	+7 (6 -> 13)	PF00026	Eukaryotic aspartyl protease	50 (50)
82070	+25 (2 -> 27)	PF00651	BTB/POZ domain	33 (28)
83501	+13 (7 -> 20)	PF00450	Serine carboxypeptidase	82 (82)
84393	-5 (5 -> 0)	PF00067	Cytochrome P450	92 (92)
86472	+5 (3 -> 8)	PF00328	Histidine phosphatase superfamily (branch 2)	40 (34)
89385	+32 (3 -> 35)	PF00075	RNase H	48 (45)
91160	+8 (2 -> 10)	PF00650	CRAL/TRIO domain	38 (31)
94341	+36 (15 -> 51)	PF13358	DDE superfamily endonuclease	195 (86)
95946	+16 (6 -> 22)	PF07690	Major Facilitator Superfamily	53 (48)
97696	+14 (8 -> 22)	PF05699	hAT family C-terminal dimerisation region	127 (2)
97817	+16 (6 -> 22)	PF00078	Reverse transcriptase (RNA-dependent DNA polymerase)	129 (68)
97889	+34 (8 -> 42)	PF09588	YqaJ-like viral recombinase domain	107 (57)
98186	+29 (11 -> 40)	PF02958	Ecdysteroid kinase	120 (117)
98261	+15 (7 -> 22)	PF00650	CRAL/TRIO domain	87 (85)
99868	+19 (11 -> 30)	PF00650	CRAL/TRIO domain	151 (137)
100866	+9 (3 -> 12)	PF03055	Retinal pigment epithelial membrane protein	52 (52)
101599	+9 (2 -> 11)	PF06585	Haemolymph juvenile hormone binding protein (JHBP)	58 (57)
39034	+4 (1 -> 5)	PF05444	Protein of unknown function (DUF753)	83 (1)
108430	+9 (4 -> 13)	PF13639	Ring finger domain	52 (37)
109223	+7 (6 -> 13)	PF09588	YqaJ-like viral recombinase domain	83 (41)
109614	+4 (2 -> 6)	PF00027	Cyclic nucleotide-binding domain	30 (20)
111466	+11 (4 -> 15)	PF00106	short chain dehydrogenase	57 (52)
111645	+3 (1 -> 4)	PF11901	Protein of unknown function (DUF3421)	24 (23)

112386	+26 (12 -> 38)	PF00858	Amiloride-sensitive sodium channel	187 (177)
112624	+15 (4 -> 19)	PF03184	DDE superfamily endonuclease	76 (49)
115371	+9 (5 -> 14)	PF00474	Sodium:solute symporter family	47 (45)
117797	+10 (10 -> 20)	PF00135	Carboxylesterase family	138 (138)
118905	+31 (9 -> 40)	PF00059	Lectin C-type domain	88 (77)
122860	+22 (4 -> 26)	PF02995	Protein of unknown function (DUF229)	67 (67)
122921	+17 (5 -> 22)	PF01130	CD36 family	61 (61)
123580	+4 (2 -> 6)	PF00589	Phage integrase family	39 (0)
123691	+6 (1 -> 7)	PF00043	Glutathione S-transferase, C-terminal domain	29 (17)
124181	+14 (7 -> 21)	PF00188	Cysteine-rich secretory protein family	75 (57)
125861	+17 (9 -> 26)	PF00892	EamA-like transporter family	109 (101)
127068	+10 (4 -> 14)	PF00106	short chain dehydrogenase	78 (75)
127634	+28 (10 -> 38)	PF05050	Methyltransferase FkbM domain	62 (57)
127702	+6 (2 -> 8)	PF13359	DDE superfamily endonuclease	32 (19)
131842	+3 (1 -> 4)	PF09588	YqaJ-like viral recombinase domain	20 (17)
131847	+19 (3 -> 22)	PF00061	Lipocalin / cytosolic fatty-acid binding protein family	38 (5)
135298	+9 (1 -> 10)	PF00060	Ligand-gated ion channel (Ionotropic glutamate receptor)	36 (6)
141959	+8 (1 -> 9)	PF00043	Glutathione S-transferase, C-terminal domain	26 (10)
143375	+8 (6 -> 14)	PF00628	PHD-finger	143 (97)
145937	+3 (1 -> 4)	PF00125	Core histone H2A/H2B/H3/H4	29 (2)
147915	+31 (11 -> 42)	PF14223	gag-polypeptide of LTR copia-type	225 (77)
150999	+16 (9 -> 25)	PF16211	C-terminus of histone H2A	184 (112)
153470	+29 (9 -> 38)	PF00125	Core histone H2A/H2B/H3/H4	131 (118)
155603	+24 (10 -> 34)	PF15511	Centromere kinetochore component CENP-T histone fold	138 (138)
158103	+8 (4 -> 12)	PF14497	Glutathione S-transferase, C-terminal domain	75 (28)
158468	+10 (9 -> 19)	PF00644	Poly(ADP-ribose) polymerase catalytic domain	159 (151)
158935	+21 (3 -> 24)	PF13975	gag-polyprotein putative aspartyl protease	49 (41)
159658	+4 (2 -> 6)	PF14291	Domain of unknown function (DUF4371)	29 (4)
160485	+3 (1 -> 4)	PF05485	THAP domain	19 (11)
166707	+4 (2 -> 6)	PF00538	linker histone H1 and H5 family	32 (29)
169474	+7 (5 -> 12)	PF09005	Domain of unknown function (DUF1897)	90 (90)
171004	+16 (2 -> 18)	-	-	-

Table S9: Gene families in *C. augens* that are significantly larger than the mean counts in insects.

clid	PFAM	description	*Dpul*	*Apis*	*Tcas*	*Dmel*	*Amel*	*Dple*	*Ocin*	*Cspl*	*Phum*	*Smar*	*Fcan*	*Caug*	*Caqu*	Mean (Insecta)	P
56848	PF08395	7tm Chemosensory receptor	1	3	4	1	1	1	6	2	1	3	2	**24**	2	1.9	6.72E-05
166091	PF13304	AAA domain, putative AbiEii toxin, Type IV TA system	0	0	0	0	0	0	0	0	0	0	0	**7**	0	0.0	8.49E-03
8640	PF00005	ABC transporter	4	5	11	10	3	5	22	6	2	9	16	**29**	14	6.0	3.19E-04
44392	PF00501	AMP-binding enzyme	2	1	1	2	1	1	2	0	1	15	1	**21**	4	1.0	8.16E-05
109398	PF16076	Acyltransferase C-terminus	1	0	1	6	1	1	2	1	1	1	1	**12**	1	1.6	5.67E-03
127634	PF01425	Amidase	1	1	1	2	1	1	1	1	1	2	4	**38**	10	1.1	1.00E-07
112235	PF00858	Amiloride-sensitive sodium channel	2	1	1	2	1	1	2	2	1	0	3	**17**	2	1.3	6.12E-04
53899	PF00155	Aminotransferase class I and II	1	2	2	2	2	2	3	2	2	2	2	**14**	3	2.0	3.70E-03
133240	PF12796	Ankyrin repeats (3 copies)	1	0	1	1	0	1	1	1	1	0	1	**11**	1	0.7	3.70E-03
68928	PF07530	Associated with zinc fingers	0	2	0	0	0	0	0	0	0	0	0	**8**	0	0.3	8.28E-03
33010	PF00651	BTB/POZ domain	1	0	1	1	0	1	1	1	1	1	1	**65**	1	0.7	0.00E+00
78766	PF00651	BTB/POZ domain	2	4	1	1	2	2	2	2	1	3	2	**14**	4	1.9	3.32E-03
118905	PF00651	BTB/POZ domain	2	0	0	6	0	3	14	10	0	1	6	**40**	6	2.7	1.00E-07
150999	PF16211	C-terminus of histone H2A	83	43	0	0	0	1	2	3	0	27	0	**25**	0	6.7	2.13E-03
122860	PF01130	CD36 family	0	1	8	9	1	2	5	2	1	2	9	**26**	1	3.4	1.14E-04
98186	PF00650	CRAL/TRIO domain	9	1	19	5	2	7	8	6	2	2	13	**40**	7	6.0	4.50E-06
98261	PF00650	CRAL/TRIO domain	5	5	7	4	5	8	5	4	6	5	10	**22**	1	5.6	2.78E-03
27628	PF00149	Calcineurin-like phosphoesterase	4	1	9	3	2	2	4	3	5	3	7	**18**	6	3.6	2.94E-03

63073	PF00149	Calcineurin-like phosphoesterase	0	3	3	5	2	5	0	5	2	0	3	**17**	6	3.6	4.16E-03
64020	PF08434	Calcium-activated chloride channel N terminal	2	7	2	6	3	5	2	5	2	2	6	**20**	4	4.3	2.47E-03
42033	PF00135	Carboxylesterase family	6	0	0	4	0	0	4	2	1	0	14	**21**	0	1.0	8.16E-05
116570	PF00135	Carboxylesterase family	1	1	1	1	1	1	1	1	1	0	1	**15**	3	1.0	1.03E-03
56529	PF00656	Caspase domain	1	3	1	1	1	1	2	1	1	4	5	**10**	2	1.3	9.49E-03
153470	PF15511	Centromere kinetochore component CENP-T histone fold	6	9	5	20	5	5	24	2	5	7	4	**38**	1	7.3	3.01E-05
40709	PF00755	Choline/Carnitine o-acyltransferase	4	3	4	7	3	4	3	10	5	4	5	**21**	8	5.1	2.96E-03
143584	PF00125	Core histone H2A/H2B/H3/H4	1	2	0	0	0	0	0	0	0	0	0	**10**	0	0.3	3.47E-03
151496	PF00125	Core histone H2A/H2B/H3/H4	0	0	0	0	0	0	0	0	0	0	0	**22**	0	0.0	1.81E-05
124181	PF00188	Cysteine-rich secretory protein family	5	12	3	11	1	3	3	3	2	2	4	**21**	5	5.0	2.75E-03
47035	PF00067	Cytochrome P450	11	23	46	25	24	24	44	19	8	10	46	**66**	11	24.1	5.46E-05
49785	PF00067	Cytochrome P450	17	5	4	3	5	5	73	17	4	8	58	**42**	14	6.1	2.30E-06
92010	PF13358	DDE superfamily endonuclease	6	0	2	1	1	1	0	2	1	2	1	**10**	2	1.1	8.49E-03
100866	PF13359	DDE superfamily endonuclease	2	1	1	1	1	3	16	2	0	3	11	**12**	1	1.3	4.32E-03
121414	PF13359	DDE superfamily endonuclease	43	289	1	0	0	0	24	44	1	4	1	**11**	2	47.9	1.09E-05
155603	PF00226	DnaJ domain	11	8	3	23	5	2	28	1	6	15	2	**34**	0	6.9	8.55E-05
164437	PF09005	Domain of unknown function (DUF1897)	1	0	1	1	1	1	1	1	1	1	1	**11**	1	0.9	4.32E-03
99868	PF14291	Domain of unknown function (DUF4371)	29	1	1	1	2	1	21	1	0	1	58	**30**	5	1.0	2.00E-06
120794	PF16064	Domain of unknown function (DUF4806)	2	0	0	0	0	1	2	0	0	0	5	**10**	1	0.1	2.99E-03

125861	PF00892	EamA-like transporter family	12	0	22	17	1	7	5	3	2	8	1	**26**	5	7.4	2.34E-03
97889	PF02958	Ecdysteroid kinase	3	35	1	1	0	0	1	19	0	0	0	**42**	5	8.0	1.09E-05
66242	PF00487	Fatty acid desaturase	0	2	1	6	1	1	8	2	1	5	6	**12**	3	2.0	8.39E-03
191185	PF00041	Fibronectin type III domain	0	0	0	0	0	0	0	0	0	0	0	**7**	0	0.0	8.49E-03
17578	PF00732	GMC oxidoreductase	0	0	1	0	1	1	27	1	1	8	0	**20**	9	0.7	8.63E-05
134686	PF17172	Glutathione S-transferase N-terminal domain	87	7	3	2	4	11	19	2	1	3	15	**23**	15	4.3	7.79E-04
122921	PF00043	Glutathione S-transferase, C-terminal domain	4	1	4	6	2	4	7	3	4	1	1	**22**	2	3.4	6.05E-04
155892	PF14497	Glutathione S-transferase, C-terminal domain	3	7	2	23	4	3	11	0	6	4	1	**30**	0	6.4	3.12E-04
158935	PF01531	Glycosyl transferase family 11	0	18	4	0	0	0	0	0	0	0	1	**24**	2	3.1	2.15E-04
83501	PF13896	Glycosyl-transferase for dystroglycan	0	6	5	5	2	7	9	5	4	2	13	**20**	4	4.9	3.42E-03
117797	PF06585	Haemolymph juvenile hormone binding protein (JHBP)	11	12	6	3	3	3	23	5	1	3	40	**20**	8	4.7	3.16E-03
53444	PF00653	Inhibitor of Apoptosis domain	11	12	25	26	6	12	8	6	3	12	16	**53**	3	12.9	5.90E-06
115371	PF00059	Lectin C-type domain	0	4	2	1	1	1	6	1	1	0	9	**14**	7	1.6	2.68E-03
118113	PF00059	Lectin C-type domain	0	0	0	0	0	0	0	0	0	0	0	**7**	0	0.0	8.49E-03
39781	PF00060	Ligand-gated ion channel	3	1	1	1	1	0	0	0	1	3	1	**37**	1	0.7	1.00E-07
53800	PF00060	Ligand-gated ion channel	2	5	2	2	3	2	3	2	2	3	6	**20**	2	2.6	6.15E-04
137329	PF00060	Ligand-gated ion channel	5	0	0	0	0	0	11	0	0	0	11	**12**	2	0.0	1.16E-03
58929	PF00917	MATH domain	12	32	0	0	0	1	0	0	0	0	0	**32**	2	4.7	3.67E-05
94341	PF07690	Major Facilitator Superfamily	0	76	5	0	0	2	1	32	1	2	15	**51**	10	16.6	1.04E-04
127655	PF05050	Methyltransferase FkbM domain	2	0	0	0	0	0	9	0	0	2	5	**13**	2	0.0	7.27E-04
59077	PF02932	Neurotransmitter-gated ion-	1	0	0	0	0	0	1	0	0	0	1	**13**	0	0.0	7.27E-04

		channel transmembrane region															
68788	PF01057	Parvovirus non-structural protein NS1	5	9	13	6	7	11	8	3	5	3	11	**31**	1	7.7	4.99E-04
15709	PF02460	Patched family	2	0	1	0	2	1	2	1	1	0	4	**15**	3	0.9	8.62E-04
157983	PF00644	Poly(ADP-ribose) polymerase catalytic domain	1	0	0	0	0	0	0	0	0	0	1	**8**	0	0.0	5.67E-03
171004	PF00644	Poly(ADP-ribose) polymerase catalytic domain	0	0	0	0	0	0	0	13	0	0	0	**18**	0	1.9	7.07E-04
160053	PF00069	Protein kinase domain	1	0	1	0	1	0	2	6	1	3	6	**10**	8	1.3	9.49E-03
127736	PF04937	Protein of unknown function (DUF 659)	2	0	0	0	0	1	0	2	0	0	0	**11**	0	0.4	2.78E-03
27531	PF06974	Protein of unknown function (DUF1298)	0	1	1	0	1	1	22	2	1	13	35	**14**	1	1.0	1.60E-03
50974	PF06974	Protein of unknown function (DUF1298)	1	1	1	1	1	1	6	1	1	2	8	**12**	1	1.0	3.32E-03
46593	PF02995	Protein of unknown function (DUF229)	0	2	1	1	1	2	0	2	1	3	0	**19**	5	1.4	3.19E-04
65583	PF02995	Protein of unknown function (DUF229)	13	1	1	0	0	0	5	1	1	7	8	**12**	4	0.6	2.28E-03
84519	PF08373	RAP domain	1	1	1	1	1	1	2	2	2	2	3	**14**	1	1.3	2.12E-03
86631	PF00075	RNase H	3	1	0	1	2	1	5	2	1	2	8	**10**	1	1.1	8.49E-03
147915	PF00075	RNase H	2	66	2	0	0	0	1	28	0	83	0	**42**	1	13.7	4.32E-04
86362	PF00355	Rieske [2Fe-2S] domain	1	1	1	1	0	0	1	1	0	0	1	**16**	1	0.6	4.32E-04
107419	PF13639	Ring finger domain	55	212	5	0	0	3	3	60	0	6	2	**15**	0	40.0	1.55E-03
108430	PF13639	Ring finger domain	1	5	1	3	2	1	14	1	1	2	6	**13**	2	2.0	5.67E-03
141366	PF00856	SET domain	1	0	0	0	0	0	0	1	0	1	0	**12**	2	0.1	1.43E-03
139416	PF12146	Serine aminopeptidase, S33	5	0	1	2	1	1	1	4	1	3	6	**12**	6	1.4	5.09E-03

82070	PF00450	Serine carboxypeptidase	0	0	0	1	1	1	1	0	1	0	1	**27**	0	0.6	4.30E-06
63522	PF05577	Serine carboxypeptidase S28	1	1	1	1	1	2	1	1	2	2	1	**12**	1	1.3	4.32E-03
113596	PF00474	Sodium:solute symporter family	16	0	0	0	0	0	8	0	0	0	0	**8**	0	0.0	5.67E-03
52512	PF00939	Sodium:sulfate symporter transmembrane region	0	0	0	3	0	0	0	0	0	0	0	**8**	0	0.4	9.33E-03
7578	PF00083	Sugar (and other) transporter	0	0	0	0	0	0	1	0	0	0	0	**7**	3	0.0	8.49E-03
13331	PF00083	Sugar (and other) transporter	2	6	8	7	4	12	8	6	5	8	10	**50**	11	6.9	1.00E-07
52239	PF00083	Sugar (and other) transporter	9	0	1	1	0	1	0	0	0	0	0	**9**	0	0.4	6.23E-03
135552	PF00685	Sulfotransferase domain	0	1	1	1	0	1	0	0	1	0	0	**13**	1	0.7	1.79E-03
139968	PF00335	Tetraspanin family	11	0	1	3	0	1	0	0	1	0	0	**13**	0	0.9	2.11E-03
215323	PF00089	Trypsin	0	0	0	0	0	0	0	0	0	0	0	**8**	0	0.0	5.67E-03
220751	PF00089	Trypsin	0	0	0	0	0	0	0	0	0	0	0	**9**	0	0.0	3.70E-03
89385	PF01663	Type I phosphodiesterase / nucleotide pyrophosphatase	0	11	0	0	0	0	0	0	0	1	1	**35**	0	1.6	4.00E-07
49470	PF00201	UDP-glucoronosyl and UDP-glucosyl transferase	7	16	5	4	2	9	9	1	0	1	19	**23**	1	5.3	1.73E-03
53356	PF00201	UDP-glucoronosyl and UDP-glucosyl transferase	6	0	3	2	2	4	6	6	1	5	11	**40**	6	2.6	1.00E-07
215795	PF02825	WWE domain	0	0	0	0	0	0	0	0	0	1	0	**7**	0	0.0	8.49E-03
127702	PF09588	YqaJ-like viral recombinase domain	2	0	1	0	1	1	18	0	0	0	0	**8**	1	0.4	9.33E-03
104662	PF11977	Zc3h12a-like Ribonuclease NYN domain	2	7	12	3	6	9	0	7	6	0	0	**22**	4	7.1	6.78E-03
42822	PF00096	Zinc finger, C2H2 type	16	136	35	15	14	17	54	23	8	20	193	**85**	12	35.4	3.59E-05
117864	PF00569	Zinc finger, ZZ type	0	2	2	3	1	1	0	1	2	0	2	**11**	0	1.7	9.36E-03
59244	PF05699	hAT family C-terminal dimerisation region	0	0	0	0	0	0	0	2	6	1	0	**11**	0	1.1	5.67E-03

95946	PF05699	hAT family C-terminal dimerisation region	4	2	8	1	2	1	1	1	1	2	2	**22**	6	2.3	2.15E-04
90160	PF00106	short chain dehydrogenase	4	2	1	0	1	0	10	1	1	3	5	**10**	5	0.9	6.39E-03
127068	PF00106	short chain dehydrogenase	1	1	1	1	2	1	22	1	2	2	28	**14**	2	1.3	2.12E-03

Table S10: Counts of Pfam domains associated with DNA photolyase in *C. augens* and 12 other arthropods.

Domain	pfamID	*Smar**	*Dpul*	*Fcan**	*Ocin*	*Caqu**	*Caug**	*Cspl*	*Apis*	*Phum*	*Amel*	*Tcas*	*Dple*	*Dmel*
DNA photolyase	PF00875	0	8	0	6	0	0	5	6	1	2	1	4	3
FAD binding domain of DNA photolyase	PF03441	0	4	0	4	0	0	3	5	1	1	1	3	2

* asterisks mark species which lack eyes.

Table S11: Counts of Pfam domains associated with the caspase domain in *C. augens* and 12 other arthropods.

Domain	pfamID	*Smar*	*Dpul*	*Fcan*	*Ocin*	*Caqu*	*Caug*	*Cspl*	*Apis*	*Phum*	*Amel*	*Tcas*	*Dple*	*Dmel*
Caspase domain	PF00656	8	18	13	14	12	35	16	6	5	5	8	4	6

Table S12: Blast hit and Pfam domains of *C. augens* EVEs.

Scaffold	Coordinates	nr best hit	e-value	Identity	Pfam hit
Scaffold_1050	14011-15100	PB1 [Wuhan Mosquito Virus 3]	3E-34	39%	PF00602 (Influenza RNA-dependent RNA polymerase subunit PB1)
Scaffold_6539	2499-3089	PB1 [Wuhan Louse Fly Virus 3]	3E-49	44%	PF00602 (Influenza RNA-dependent RNA polymerase subunit PB1)
Scaffold_12940	1460-1897	PB1 [Wuhan Mosquito Virus 5]	5E-48	51%	PF00602 (Influenza RNA-dependent RNA polymerase subunit PB1)
Scaffold_5450	15766-16765	PB1 [Wuhan Louse Fly Virus 3]	1E-25	43%	PF00602 (Influenza RNA-dependent RNA polymerase subunit PB1)
Scaffold_240	94436-94965	PB1 [Wuhan Mosquito Virus 3]	8E-32	42%	-
Scaffold_2171	26421-27126	PB1 [Shuangao Insect Virus 4]	2E-06	39%	-
Scaffold_2171	22958-23373	polymerase basic 1 protein [Wellfleet Bay virus]	3E-07	33%	-

Table S13: Accession number of PB1 proteins from orthomyxoviruses used in the phylogenetic analysis of *C. augens* EVEs.

Virus	PB1 Genbank Accesion
Influenzavirus A	BAU68348.1
Influenzavirus B	NP_056657.1
Influenzavirus C	YP_089653.1
Influenzavirus D	AIE52114.1
Salmon_isavirus	YP_145804.1
Quaranfil quaranjavirus	ACY56282.1
Johnston Atoll virus	ACY56284.1
Wellfleet Bay virus	YP_009110686.1
Aransas bay virus	AHB34061.1
Dhori thogotovirus	ADF56030.1
Thogoto thogotovirus	YP_145794.1
Jingshan Fly Virus 1	AJG39084.1
Sanxia Water Strider Virus 3	AJG39086.1
Shuangao Insect Virus 4	AJG39088.1
Wuhan Louse Fly Virus 3	AJG39089.1
Wuhan Louse Fly Virus 4	AJG39090.1
Wuhan Mosquito Virus 3	AJG39091.1
Wuhan Mosquito Virus 4	AJG39092.1
Wuhan Mosquito Virus 5	AJG39093.1
Wuhan Mosquito Virus 6	AJG39094.1
Wuhan Mosquito Virus 7	AJG39095.1
Wuhan Mothfly Virus	AJG39096.1
Sinu virus	APP91612.1

Table S14: Source of genomic data used in the analyses.

Species	Fasta	gff	Protein
caqu	https://i5k.nal.usda.gov/sites/default/files/data/Arthropoda/cataqu-%28Catajapyx_aquilonaris%29/Current%20Genome%20Assembly/1.Genome%20Assembly/BCM-After-Atlas/Scaffolds/forcepstail.consistent.scaffolds.fa.gz	https://i5k.nal.usda.gov/sites/default/files/data/Arthropoda/cataqu-%28Catajapyx_aquilonaris%29/Current%20Genome%20Assembly/2.Official%20or%20Primary%20Gene%20Set/BCM_version_0.5.3/consensus_gene_set/CAQU.Models.gff3.gz	https://i5k.nal.usda.gov/sites/default/files/data/Arthropoda/cataqu-%28Catajapyx_aquilonaris%29/Current%20Genome%20Assembly/2.Official%20or%20Primary%20Gene%20Set/BCM_version_0.5.3/consensus_gene_set/CAQU.faa.gz
fcan	ftp://ftp.ncbi.nlm.nih.gov/genomes/refseq/invertebrate/Folsomia_candida/representative/GCF_002217175.1_ASM221717v1/GCF_002217175.1_ASM221717v1_genomic.fna.gz	ftp://ftp.ncbi.nlm.nih.gov/genomes/refseq/invertebrate/Folsomia_candida/representative/GCF_002217175.1_ASM221717v1/GCF_002217175.1_ASM221717v1_genomic.gff.gz	ftp://ftp.ncbi.nlm.nih.gov/genomes/refseq/invertebrate/Folsomia_candida/representative/GCF_002217175.1_ASM221717v1/GCF_002217175.1_ASM221717v1_protein.faa.gz
ocin	ftp://ftp.ncbi.nlm.nih.gov/genomes/genbank/invertebrate/Orchesella_cincta/representative/GCA_001718145.1_ASM171814v1/GCA_001718145.1_ASM171814v1_genomic.fna.gz	ftp://ftp.ncbi.nlm.nih.gov/genomes/genbank/invertebrate/Orchesella_cincta/representative/GCA_001718145.1_ASM171814v1/GCA_001718145.1_ASM171814v1_genomic.gff.gz	ftp://ftp.ncbi.nlm.nih.gov/genomes/genbank/invertebrate/Orchesella_cincta/representative/GCA_001718145.1_ASM171814v1/GCA_001718145.1_ASM171814v1_protein.faa.gz
dpul	ftp://ftp.ncbi.nlm.nih.gov/genomes/genbank/invertebrate/Daphnia_pulex/representative/GCA_000187875.1_V1.0/GCA_000187875.1_V1.0_genomic.fna.gz	ftp://ftp.ncbi.nlm.nih.gov/genomes/genbank/invertebrate/Daphnia_pulex/representative/GCA_000187875.1_V1.0/GCA_000187875.1_V1.0_genomic.gff.gz	ftp://ftp.ncbi.nlm.nih.gov/genomes/genbank/invertebrate/Daphnia_pulex/representative/GCA_000187875.1_V1.0/GCA_000187875.1_V1.0_protein.faa.gz
smar	ftp://ftp.ensemblgenomes.org/pub/metazoa/release-38/fasta/strigamia_maritima/dna/Strigamia_maritima.Smar1.dna.toplevel.fa.gz	ftp://ftp.ensemblgenomes.org/pub/metazoa/release-38/gff3/strigamia_maritima/Strigamia_maritima.Smar1.38.gff3.gz	ftp://ftp.ensemblgenomes.org/pub/metazoa/release-38/fasta/strigamia_maritima/pep/Strigamia_maritima.Smar1.pep.all.fa.gz
apis	ftp://ftp.ensemblgenomes.org/pub/metazoa/release-38/fasta/acyrthosiphon_pisum/dna/Acyrthosiphon_pisum.Acyr_2.0.dna.toplevel.fa.gz	ftp://ftp.ensemblgenomes.org/pub/metazoa/release-38/gff3/acyrthosiphon_pisum/Acyrthosiphon_pisum.Acyr_2.0.38.gff3.gz	ftp://ftp.ensemblgenomes.org/pub/metazoa/release-38/fasta/acyrthosiphon_pisum/pep/Acyrthosiphon_pisum.Acyr_2.0.pep.all.fa.gz
phum	https://www.vectorbase.org/download/pediculus-humanus-usdascaffoldsphumu2fagz	https://www.vectorbase.org/download/pediculus-humanus-usdabasefeaturesphumu24gff3gz	https://www.vectorbase.org/download/pediculus-humanus-usdapeptidesphumu24fagz
amel	ftp://ftp.ncbi.nlm.nih.gov/genomes/refseq/invertebrate/Apis_mellifera/representative/GCF_000002195.4_Amel_4.5/	ftp://ftp.ncbi.nlm.nih.gov/genomes/refseq/invertebrate/Apis_mellifera/representative/GCF_000002195.4_Amel_4.5/	ftp://ftp.ncbi.nlm.nih.gov/genomes/refseq/invertebrate/Apis_mellifera/representative/GCF_000002195.4_Amel_4.5/

	GCF_000002195.4_Amel_4.5_genomic.fna.gz	GCF_000002195.4_Amel_4.5_genomic.gff.gz	GCF_000002195.4_Amel_4.5_protein.faa.gz
tcas	ftp://ftp.ncbi.nlm.nih.gov/genomes/refseq/invertebrate/Tribolium_castaneum/representative/GCF_000002335.3_Tcas5.2/GCF_000002335.3_Tcas5.2_genomic.fna.gz	ftp://ftp.ncbi.nlm.nih.gov/genomes/refseq/invertebrate/Tribolium_castaneum/representative/GCF_000002335.3_Tcas5.2/GCF_000002335.3_Tcas5.2_genomic.gff.gz	ftp://ftp.ncbi.nlm.nih.gov/genomes/refseq/invertebrate/Tribolium_castaneum/representative/GCF_000002335.3_Tcas5.2/GCF_000002335.3_Tcas5.2_protein.faa.gz
dple	ftp://ftp.ensemblgenomes.org/pub/metazoa/release-38/fasta/danaus_plexippus/dna/Danaus_plexippus.Dpv3.dna.toplevel.fa.gz	ftp://ftp.ensemblgenomes.org/pub/metazoa/release-38/gff3/danaus_plexippus/Danaus_plexippus.Dpv3.38.gff3.gz	ftp://ftp.ensemblgenomes.org/pub/metazoa/release-38/fasta/danaus_plexippus/pep/Danaus_plexippus.Dpv3.pep.all.fa.gz
dmel	ftp://ftp.flybase.net/genomes/Drosophila_melanogaster/current/fasta/dmel-all-chromosome-r6.22.fasta.gz<br><br>ftp://ftp.ncbi.nlm.nih.gov/genomes/refseq/invertebrate/Drosophila_melanogaster/reference/GCF_000001215.4_Release_6_plus_ISO1_MT/GCF_000001215.4_Release_6_plus_ISO1_MT_genomic.fna.gz	ftp://ftp.flybase.net/genomes/Drosophila_melanogaster/current/gff/dmel-all-filtered-r6.22.gff.gz<br><br>ftp://ftp.ncbi.nlm.nih.gov/genomes/refseq/invertebrate/Drosophila_melanogaster/reference/GCF_000001215.4_Release_6_plus_ISO1_MT/GCF_000001215.4_Release_6_plus_ISO1_MT_genomic.gff.gz	ftp://ftp.flybase.net/genomes/Drosophila_melanogaster/current/fasta/dmel-all-translation-r6.22.fasta.gz<br><br>ftp://ftp.ncbi.nlm.nih.gov/genomes/refseq/invertebrate/Drosophila_melanogaster/reference/GCF_000001215.4_Release_6_plus_ISO1_MT/GCF_000001215.4_Release_6_plus_ISO1_MT_protein.faa.gz
lmig	ftp://ftp.ncbi.nlm.nih.gov/genomes/genbank/invertebrate/Locusta_migratoria/representative/GCA_000516895.1_LocustGenomeV1/GCA_000516895.1_LocustGenomeV1_genomic.fna.gz		
bger	ftp://ftp.ncbi.nlm.nih.gov/genomes/genbank/invertebrate/Blattella_germanica/representative/GCA_003018175.1_Bger_1.1/GCA_003018175.1_Bger_1.1_genomic.fna.gz		
choo	ftp://ftp.ncbi.nlm.nih.gov/genomes/genbank/invertebrate/Clitarchus_hookeri/representative/GCA_002778355.1_ASM277835v1/GCA_002778355.1_ASM277835v1_genomic.fna.gz		
mext	ftp://ftp.ncbi.nlm.nih.gov/genomes/genbank/invertebrate/Medauroidea_extradentata/representative/GCA_003012365.1_ASM301236v1/GCA_003012365.1_ASM301236v1_genomic.fna.gz		

SUPPLEMENTARY FIGURES

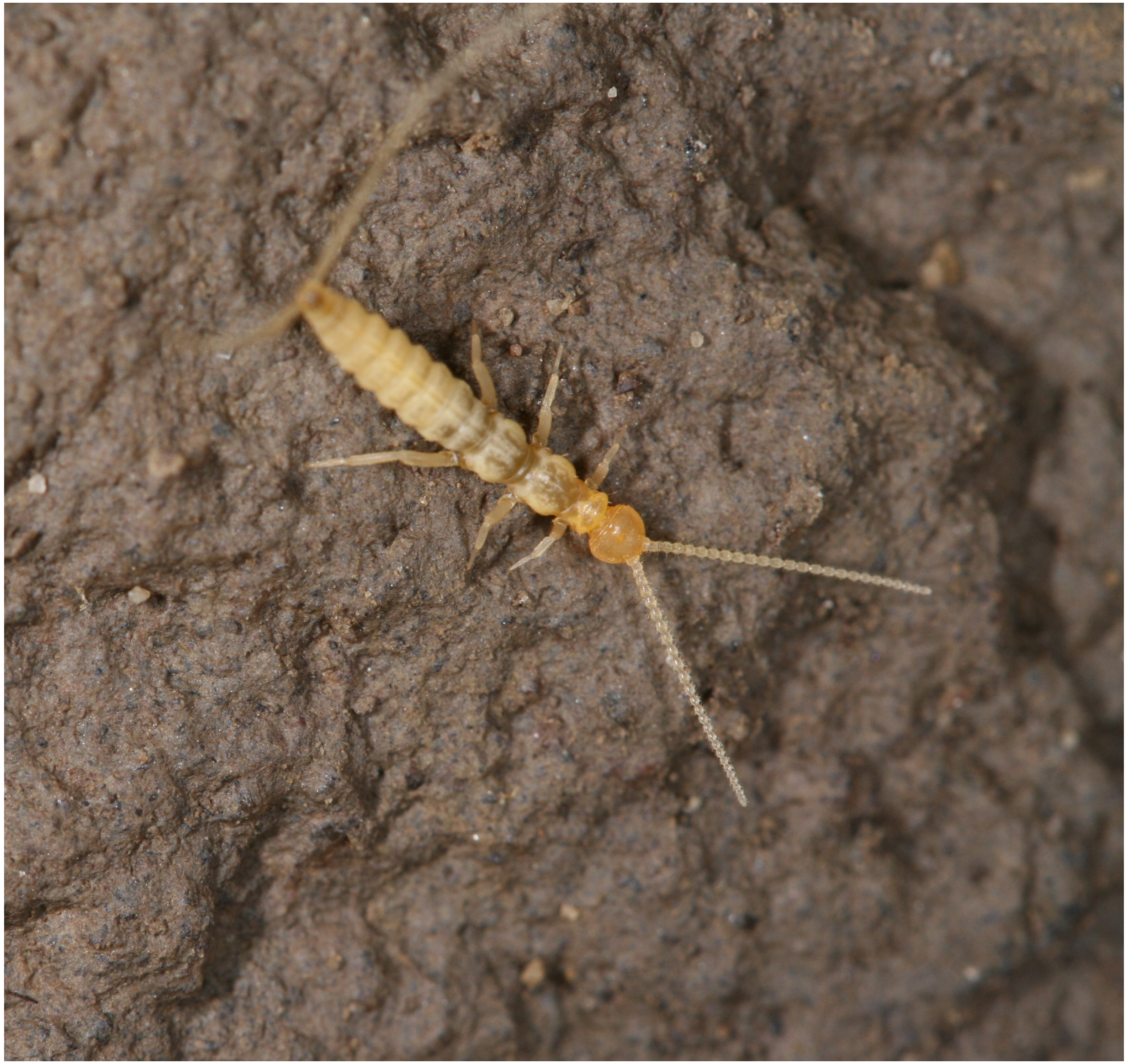

Figure S1a: High-resolution picture of *C. augens*.

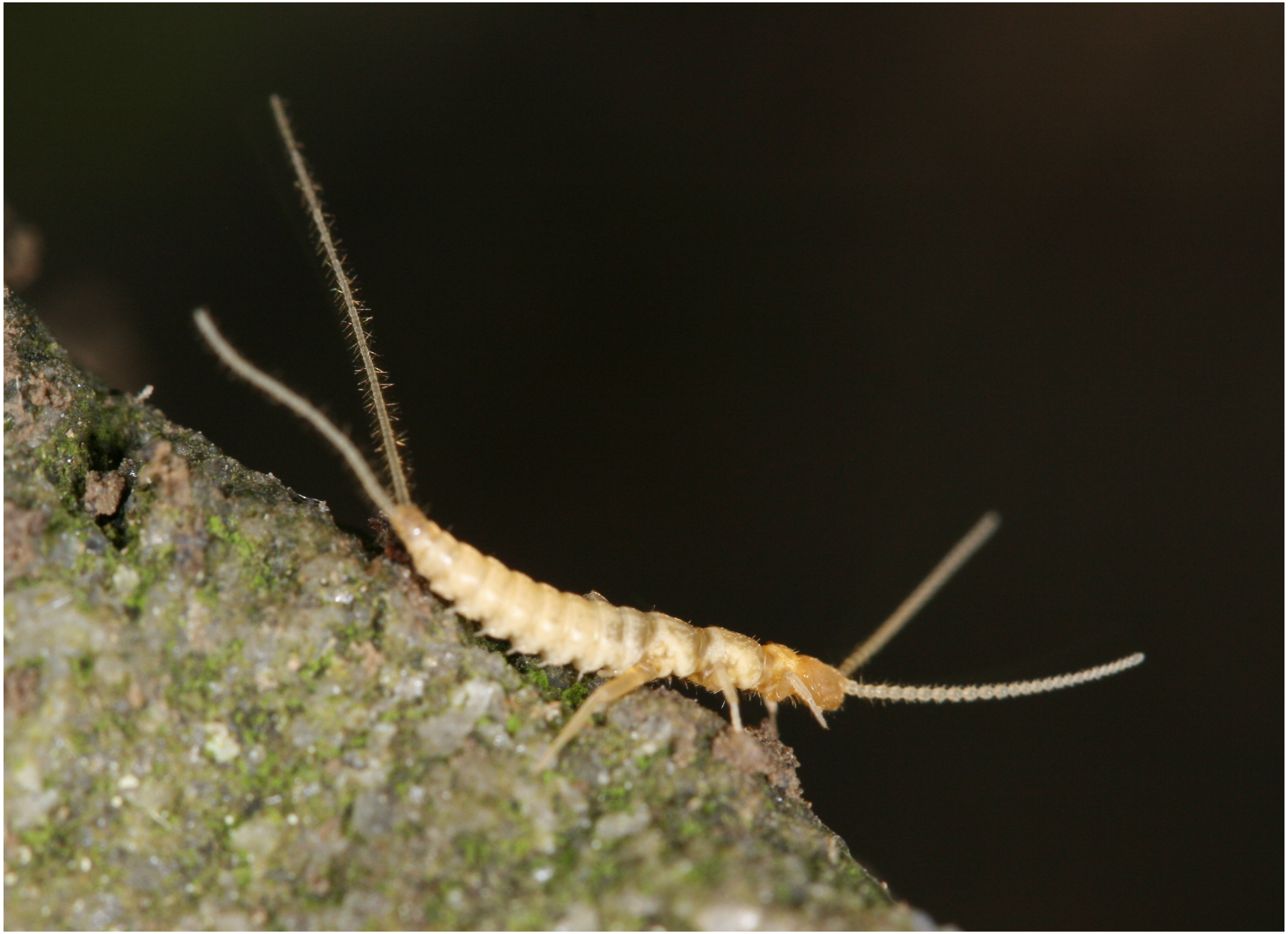

Figure S1b: High-resolution picture of *C. augens*.

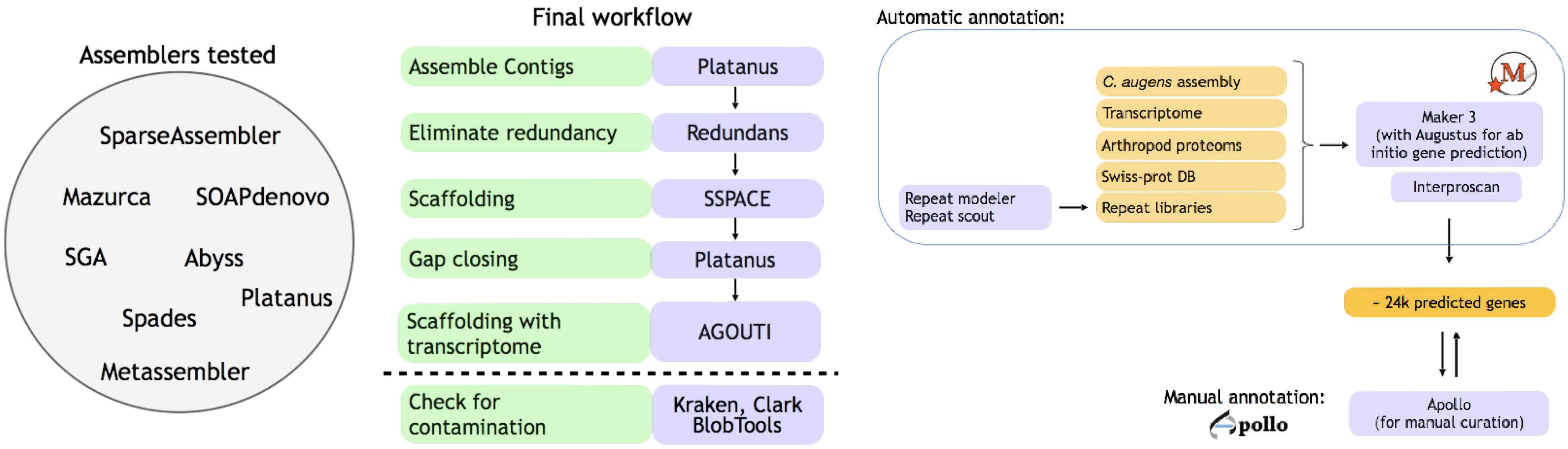

Figure S2: Assembly and annotation workflow used for *C. augens*.

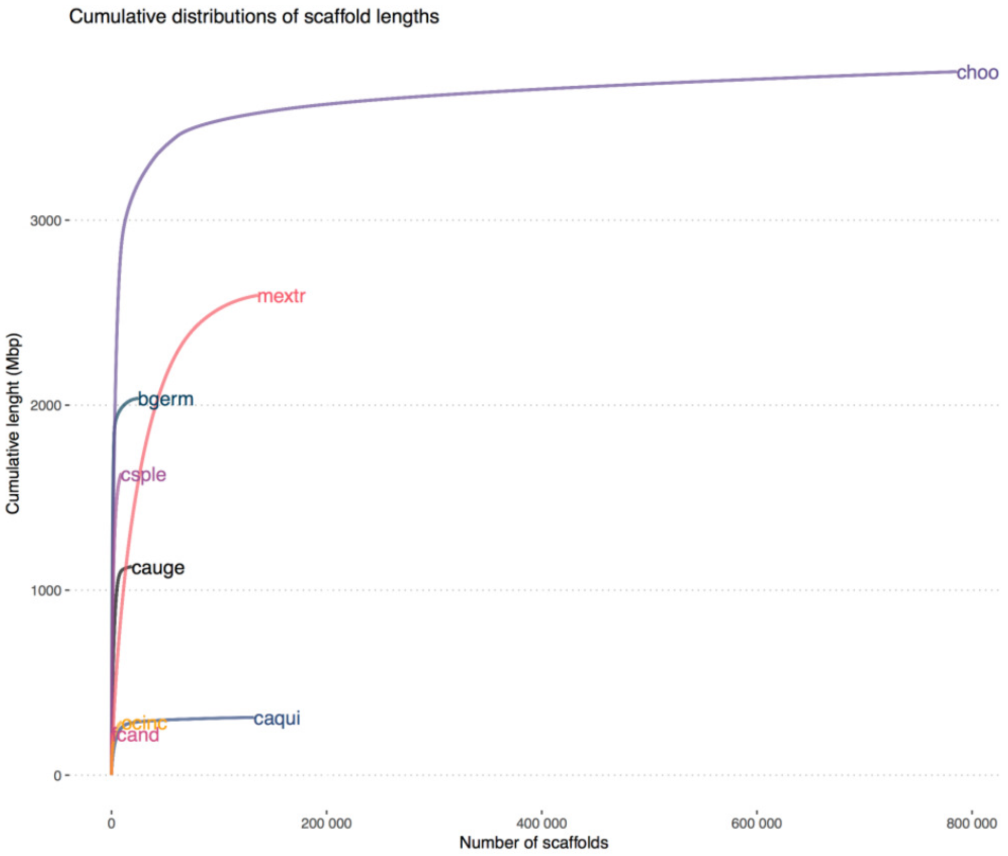

Figure S3: Cumulative distributions of scaffold lengths in *C. augens* and other arthropods. Csple, *Calopteryx splendens* (banded demoiselle); Caqui, *Catajapyx aquilonaris* (northern forcepstail); Fcand, *Folsomia candida* (springtail); Ocinc, *Orchesella cincta* (springtail); Bgerm, *Blattella germanica*; Chook, *Clitarchus hookeri*; Mextr, *Medauroidea extradentata*.

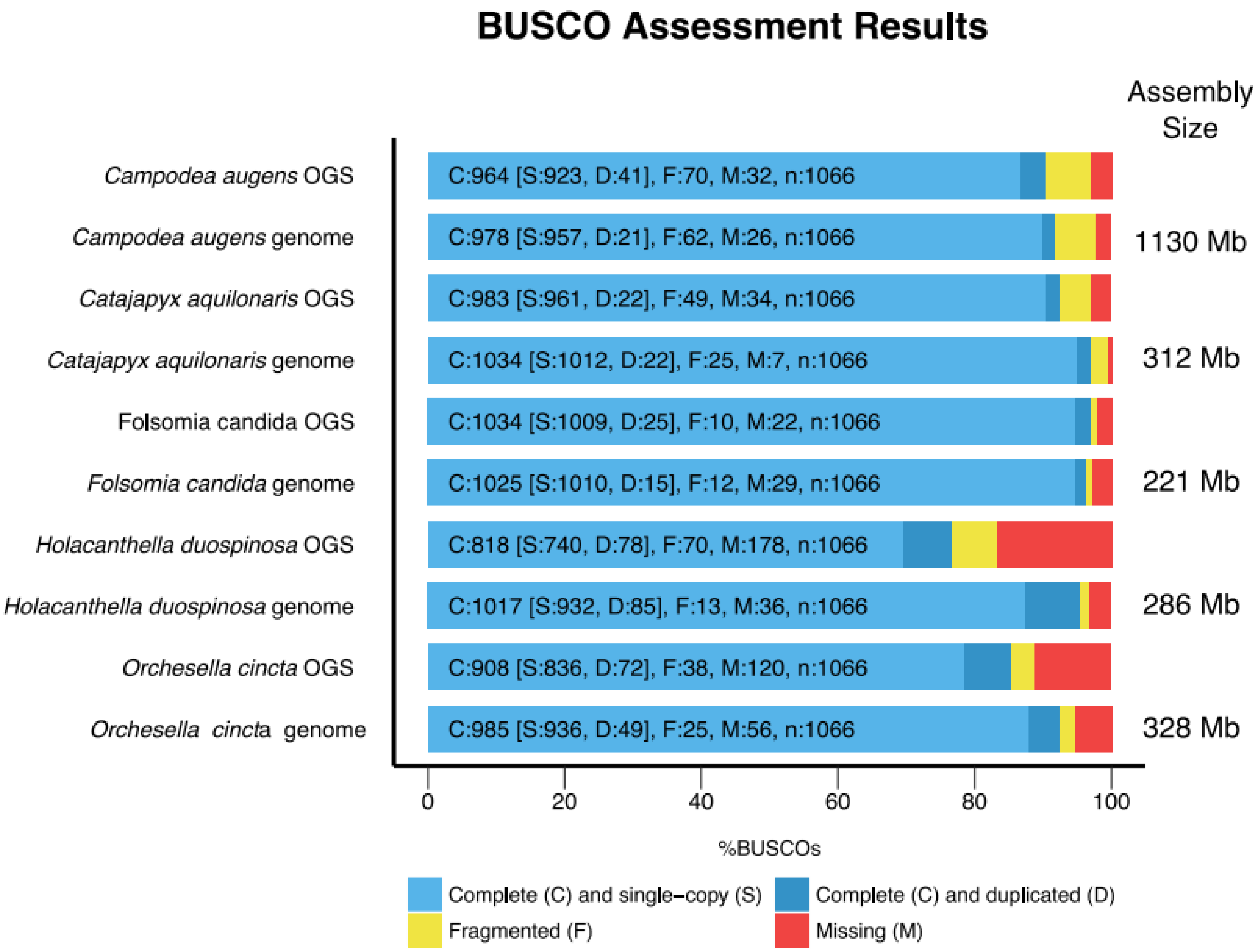

Figure S4: Plots of BUSCO scores for *C. augens* and the other ancestrally wingless hexapods using the arthropoda_v9 dataset.

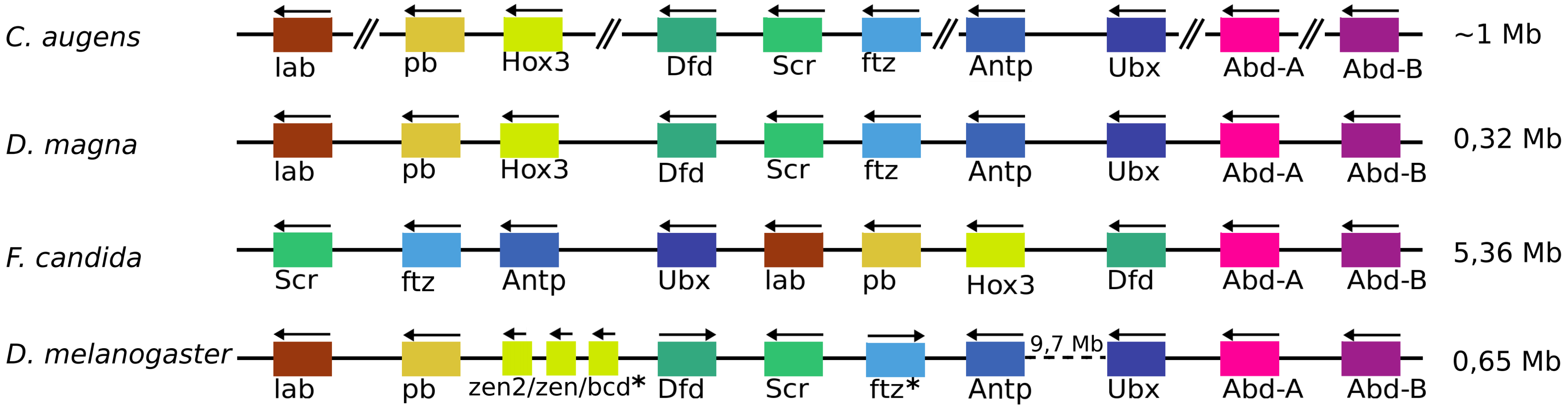

Figure S5: Hox genes cluster arrangement for *Campodea augens, Daphnia magna, Folsomia candida* and *Drosophila melanogaster*.
Double slash delimits different scaffolds. Dotted line indicates a large genomic region of ca. 10 Mb which characterizes the hox genes cluster of *D. melanogaster*.
(*) in *D. melanogaster* genes indicates genes that do not have homeotic function (Hughes & Kaufman 2002).

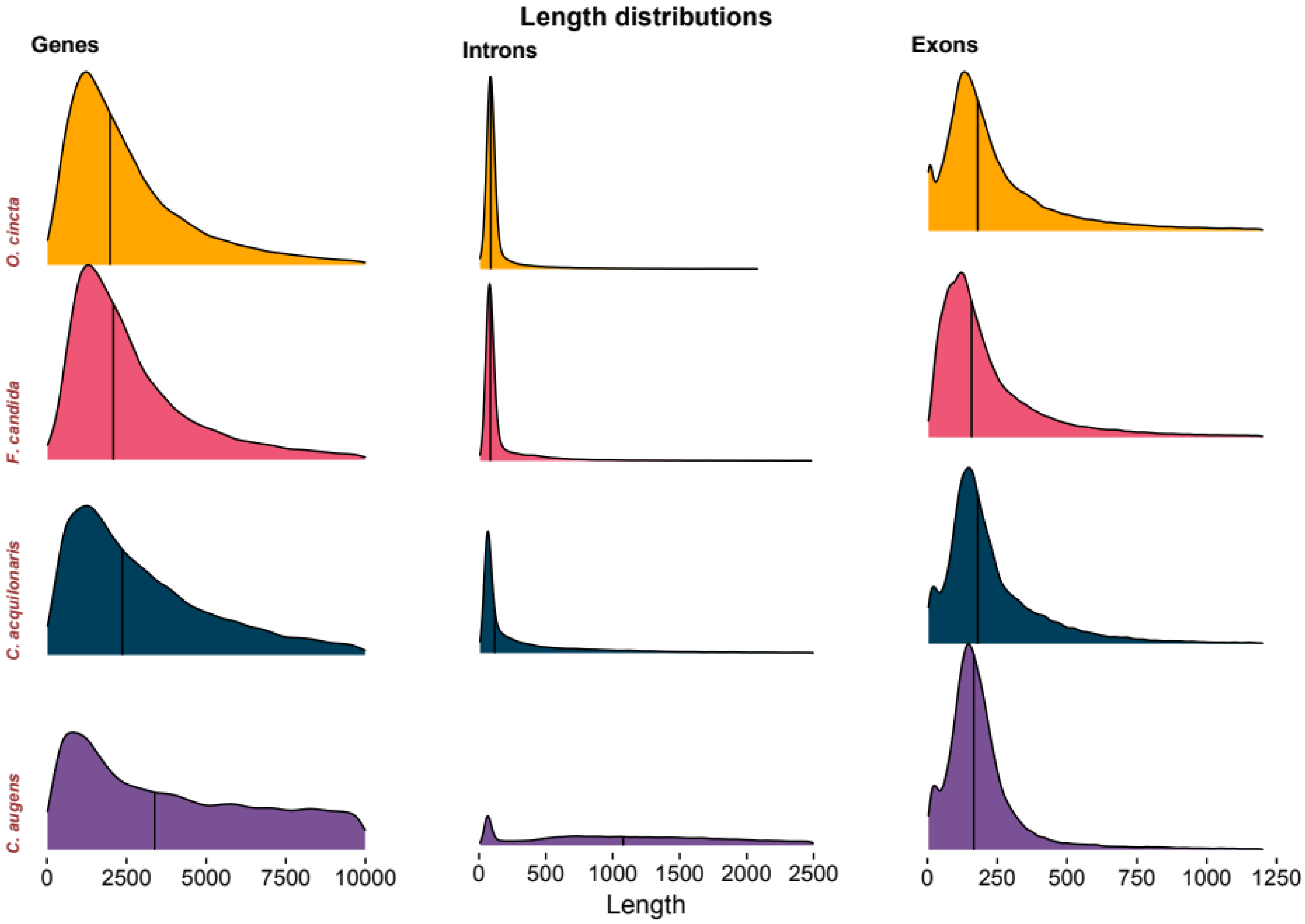

Figure S6: Gene, intron and exon length distributions in *C. augens* and other ancestrally wingless hexapods.

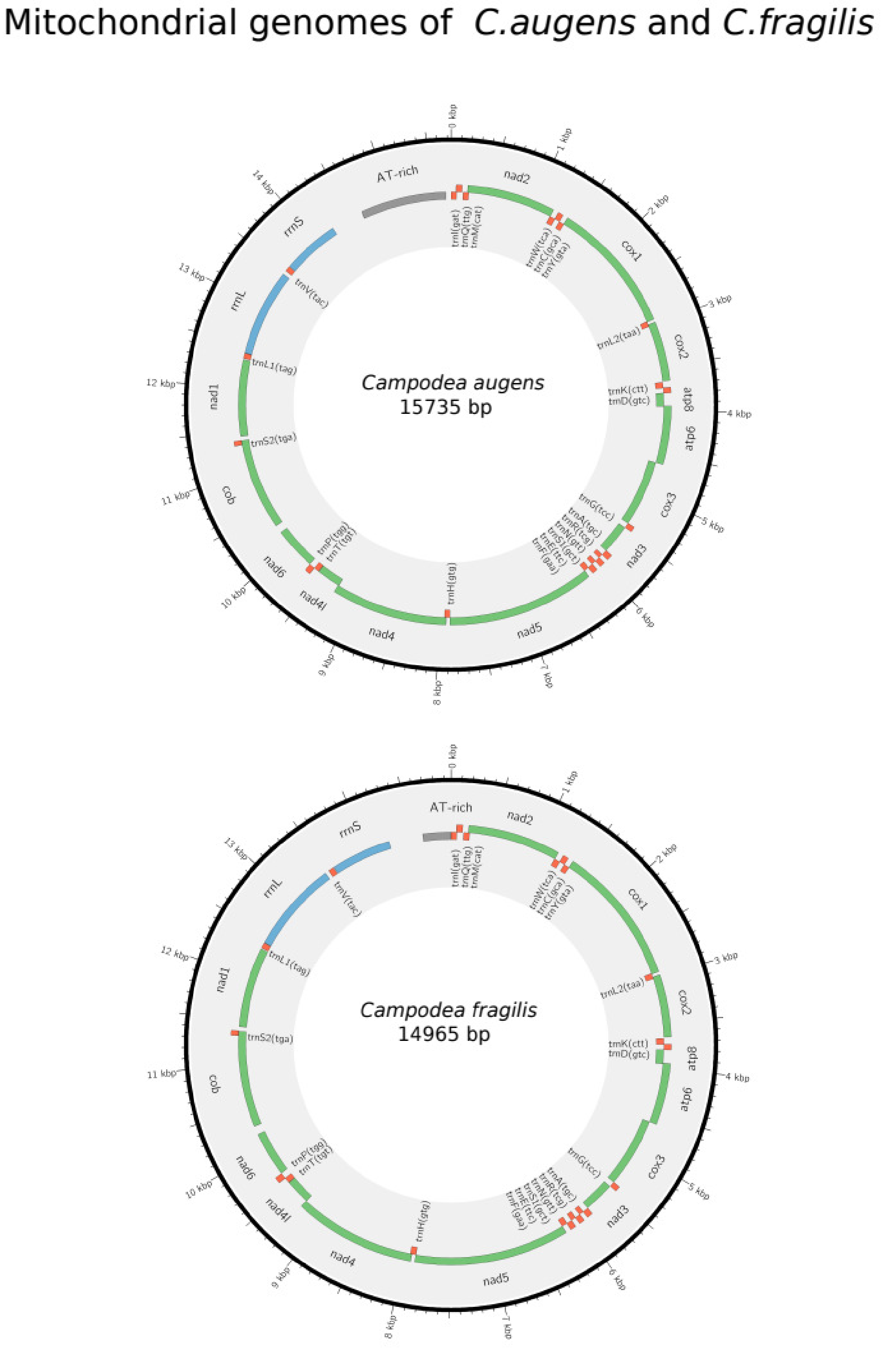

Figure S7: Graphical representation of the mitochondrial genomes of *C. augens* and *C. fragilis.*

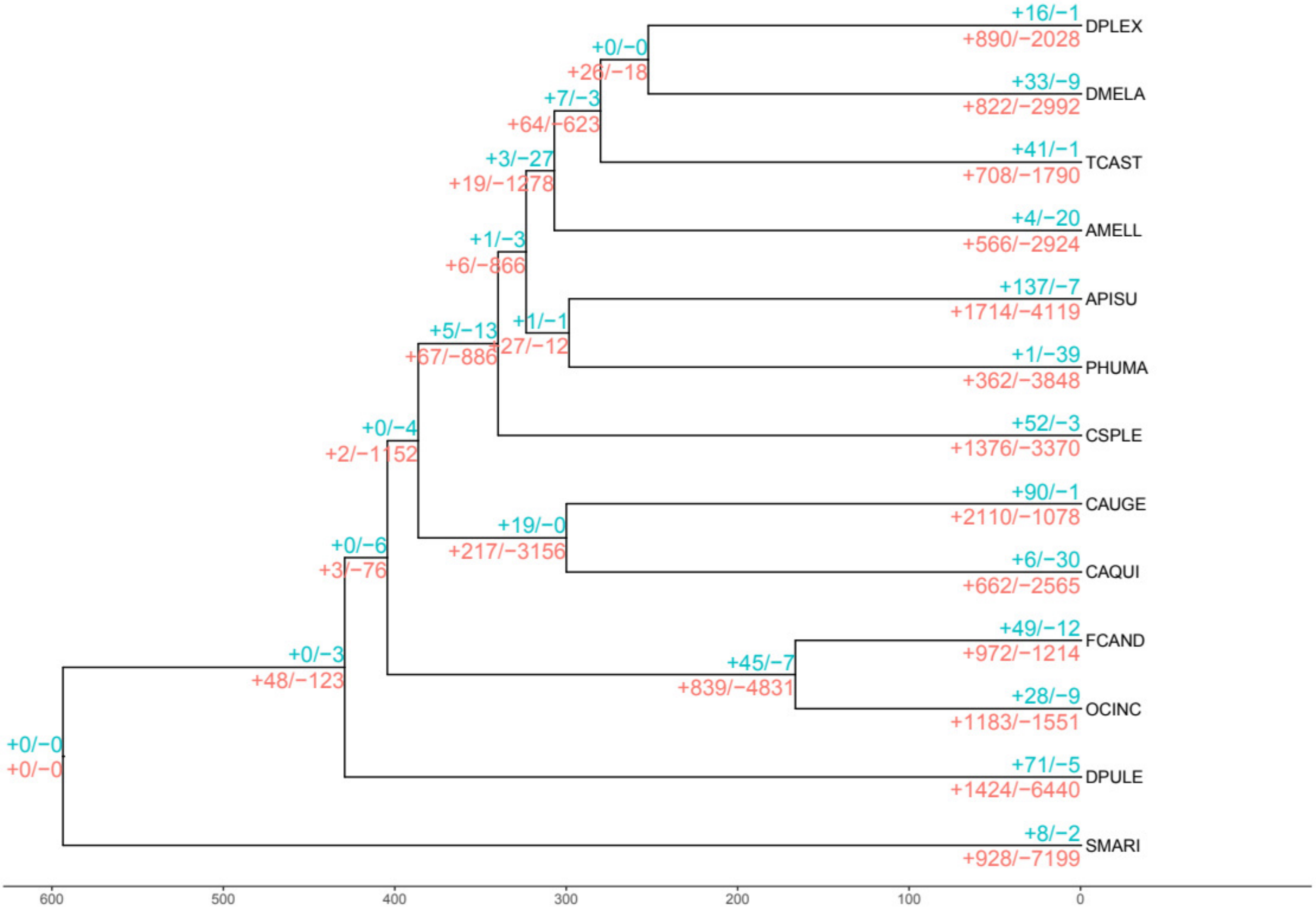

Figure S8: Ultrametric species phylogeny displaying expansions/contractions of gene families as estimated using CAFE. Values in green correspond to the number of significantly (Viterbi p-value < 0.01) expanded (+) and contracted (-) gene families. Values in red correspond to the total number of expansions/contractions including the non-significant ones, and correspond to the values represented in the pie charts of Figure 2.

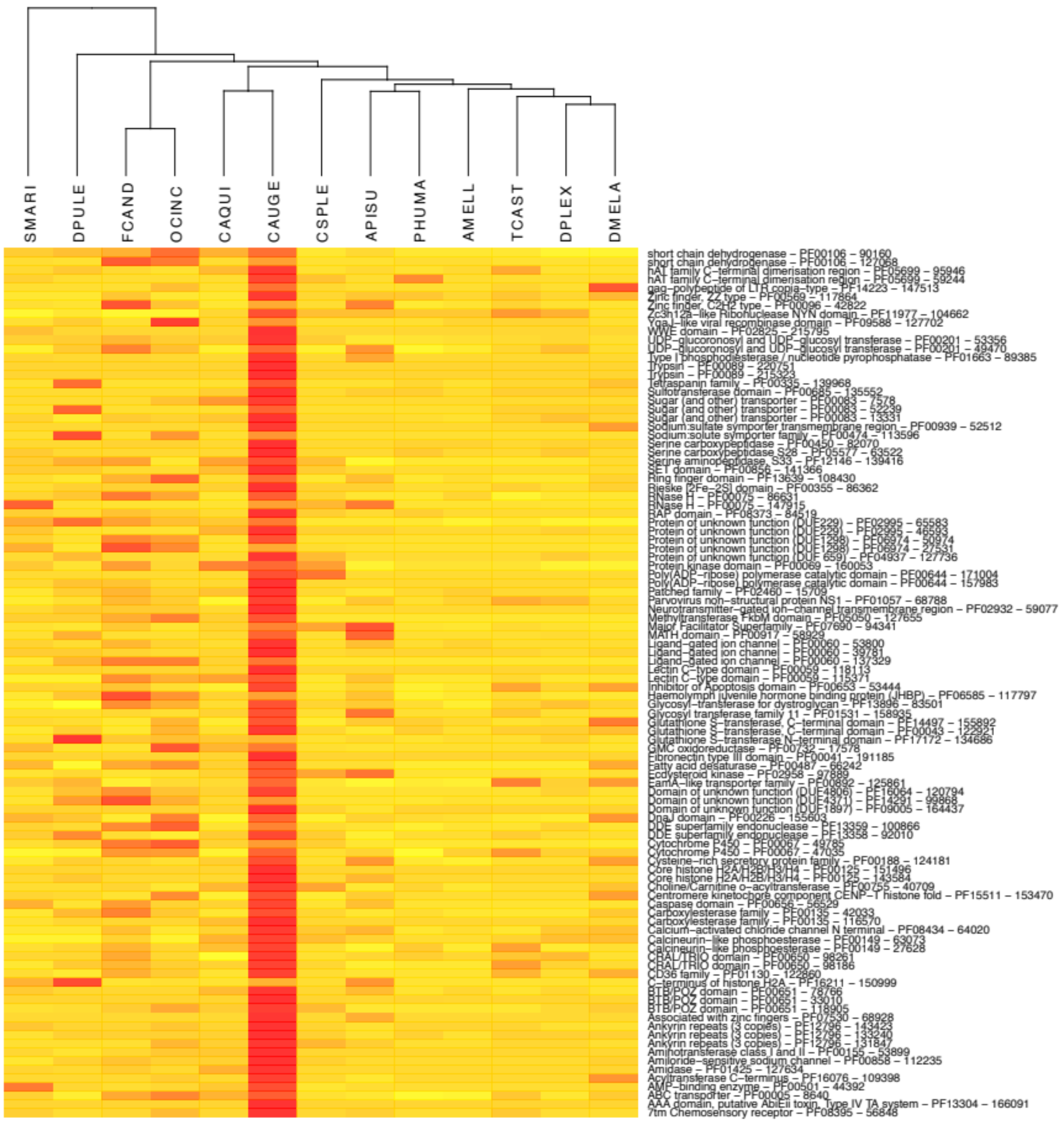

Figure S9: Heat map of OrthoDB clusters that are significantly larger in *C. augens* compared to the mean counts from insect species. The most representative pfam domain for each cluster is reported on the right.

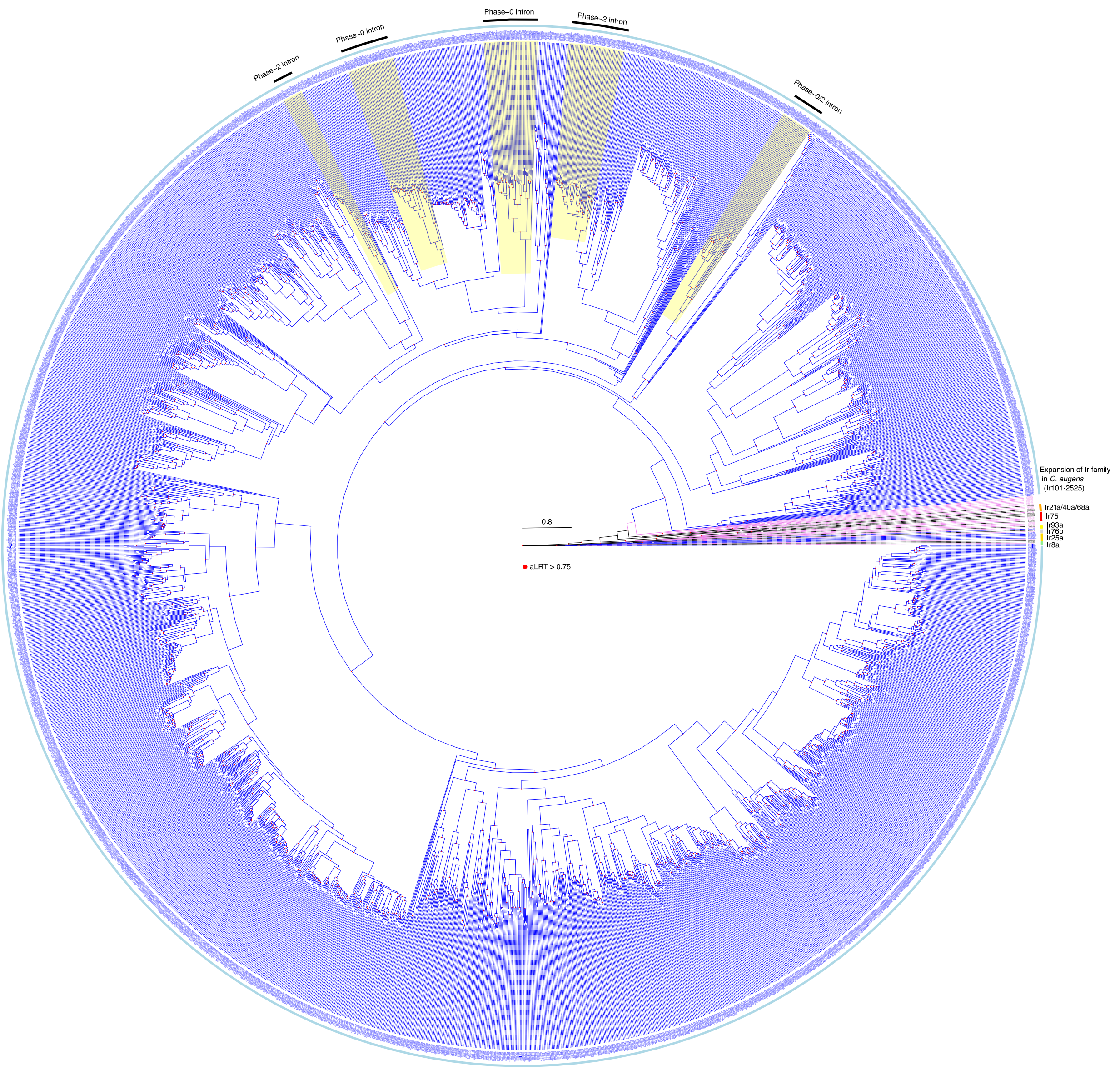

Figure S10: Phylogenetic tree of the IR family. This tree was rooted by declaring the Ir8a and 25a lineages as the outgroup, based on their basal positions within larger trees including the ionotropic glutamate receptors from which the IRs evolved. The *Campodea augens* (Caug) proteins are in blue, the *Drosophila melanogaster* (Dmel) proteins for the seven conserved IRs with orthologs in *C. augens*, as well as the Ir75 clade, are colored black, while the *Calopteryx splendens* (Cspl) proteins are colored purple. The seven conserved lineages are highlight in colors and indicated outside the circle. The five CaugIr lineages that have idiosyncratically gained introns are highlighted in yellow, with the intron phase(s) indicated outside the circle, showing the independence of these five lineages whose introns are all in different locations. The scale bar indicates substitutions per site. Red filled circles indicate nodes with an approximate Likelihood-Ratio Test (aLRT) > 0.75. Zoom in to view the details and names of proteins.

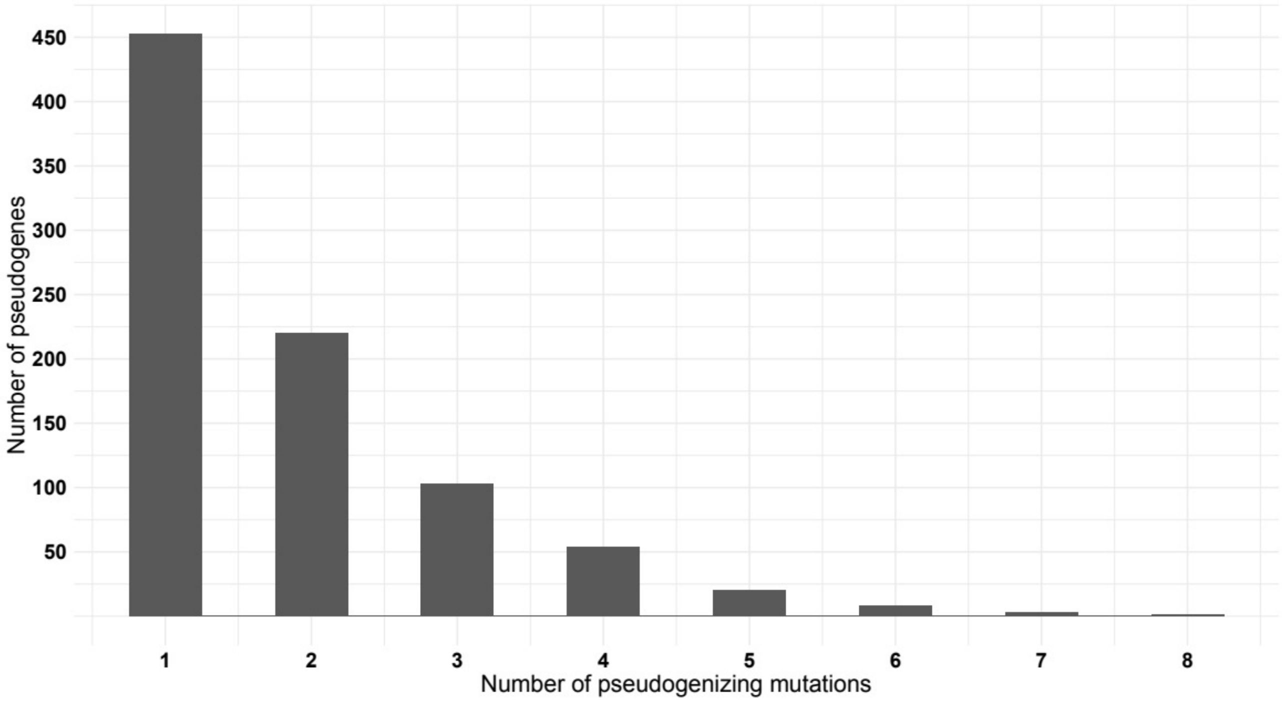

Figure S11: Histogram of the numbers of pseudogenes with 1-8 pseudogenizing mutations.

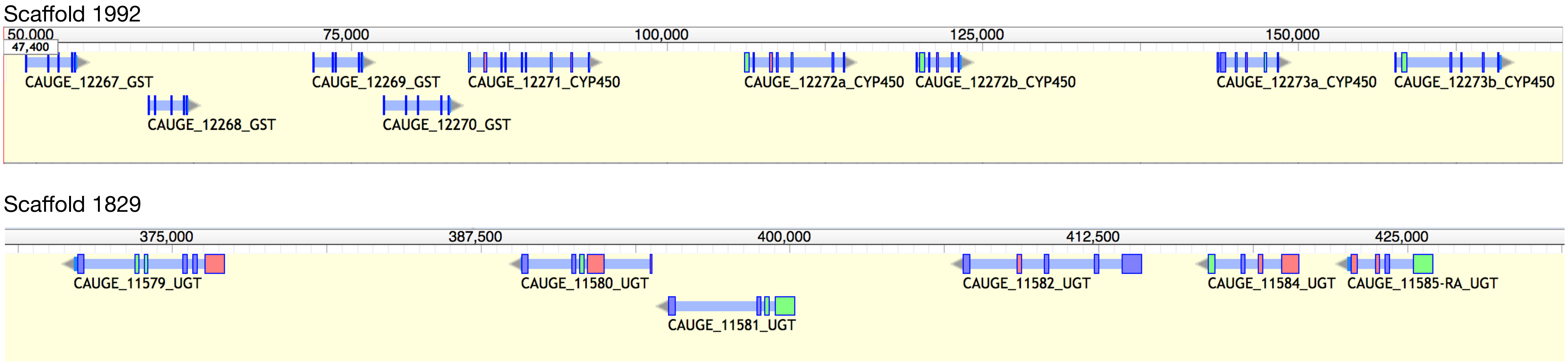

Figure S12: Genomic regions encoding a cluster of 5 CYP450 with 4 GST genes (Scaffold_1992), and a cluster of 6 UGTs (Scaffold_1829). Large boxes correspond to exons.

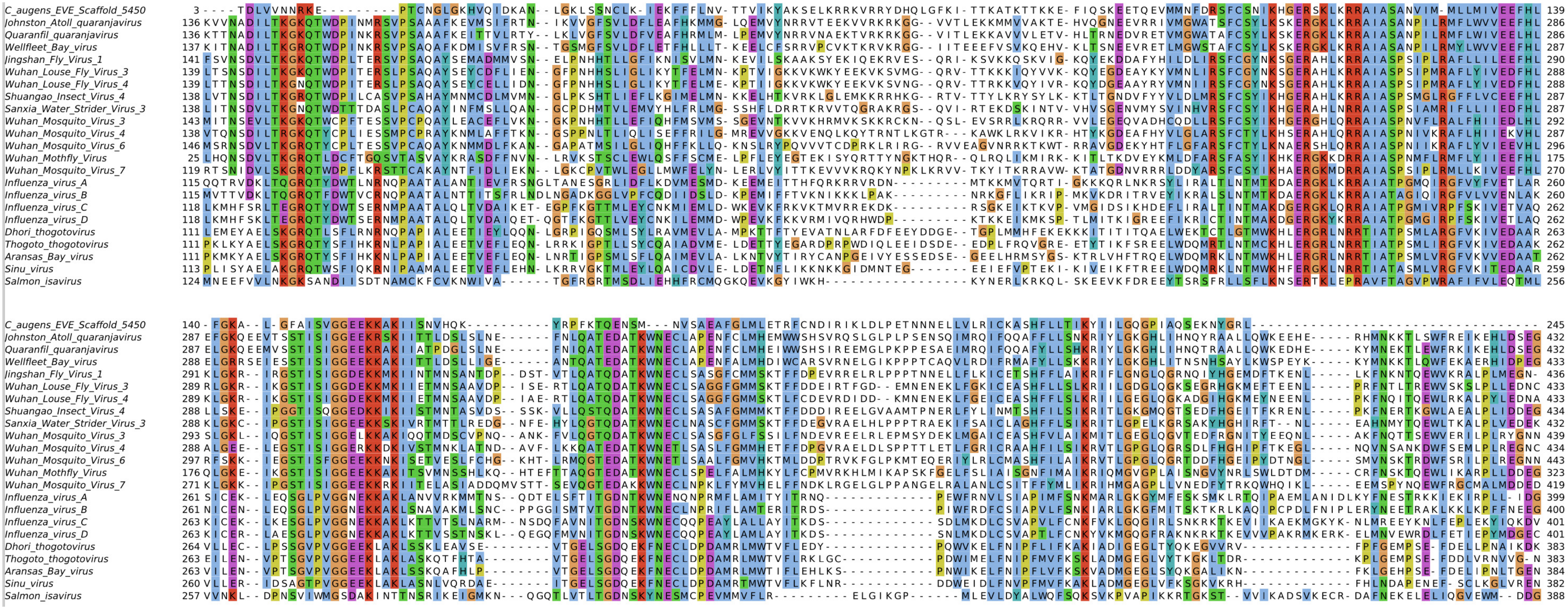

Figure S13: MSA of a putative endogenous viral elements in *C. augens* genome (C_augens_EVE_5450) and PB1 sequences of Orthomyxoviridae.

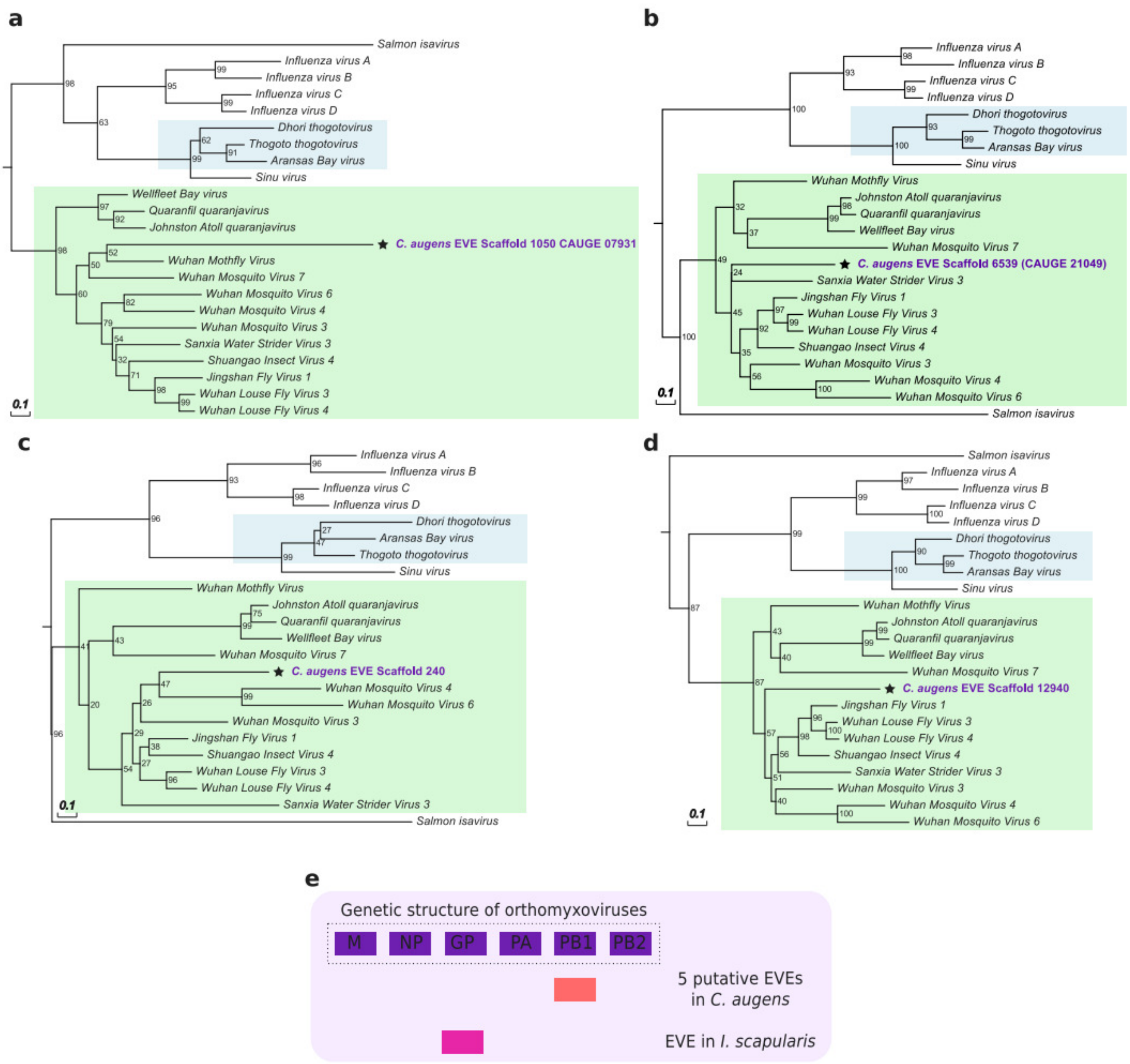

Figure S14: Phylogenetic relationships of the EVEs found in *C. augens* related to -ssRNA viruses of the Orthomyxoviridae family. **a-d**, All EVEs in *C. augens* correspond to orthomyxoviral Polymerase Basic protein 1 (PB1) (Pfam Id PF00602; "Flu_PB1"). Neighbor-joining trees were constructed using alignments of EVE amino acid sequences with BP1 proteins of representatives of the Orthomyxoviridae family (see Supplementary Table 14 for GenBank accession nos.). Support for trees was evaluated using 1,000 pseudo replicates. Values correspond to the bootstrap support values. Scale bars indicate amino acid substitutions per site. Green boxes highlight viruses of the Quaranjavirus genus. **e**, Comparison of EVEs related to quaranjaviruses found in *C. augens* and the tick *Ixodes scapularis* (Katzourakis and Gifford, 2010). While all EVEs in *C. augens* correspond to PB1, EVE in *I. scapularis* corresponds to the glycoprotein (GP) of quaranjaviruses.

REFERENCES

Bernt M et al. 2013. MITOS: Improved de novo metazoan mitochondrial genome annotation. Molecular Phylogenetics and Evolution. 69:313–319. doi: 10.1016/j.ympev.2012.08.023.

Darriba D, Taboada GL, Doallo R, Posada D. 2011. ProtTest 3: fast selection of best-fit models of protein evolution. Bioinformatics. 27:1164–1165. doi: 10.1093/bioinformatics/btr088.

Dierckxsens N, Mardulyn P, Smits G. 2017. NOVOPlasty: de novo assembly of organelle genomes from whole genome data. Nucleic Acids Res. 45:e18–e18. doi: 10.1093/nar/gkw955.

Grabherr MG et al. 2011. Trinity: reconstructing a full-length transcriptome without a genome from RNA-Seq data. Nat. Biotechnol. 29:644–652. doi: 10.1038/nbt.1883.

Hughes CL, Kaufman TC. 2002. Hox genes and the evolution of the arthropod body plan1. Evol. Dev. 4:459–499. doi: 10.1046/j.1525-142X.2002.02034.x.

Katoh K, Standley DM. 2013. MAFFT Multiple Sequence Alignment Software Version 7: Improvements in Performance and Usability. Mol. Biol. Evol. 30:772–780. doi: 10.1093/molbev/mst010.

Kriventseva EV et al. 2019. OrthoDB v10: sampling the diversity of animal, plant, fungal, protist, bacterial and viral genomes for evolutionary and functional annotations of orthologs. Nucleic Acids Res. 47:D807–D811. doi: 10.1093/nar/gky1053.

Nguyen L-T, Schmidt HA, von Haeseler A, Minh BQ. 2015. IQ-TREE: A Fast and Effective Stochastic Algorithm for Estimating Maximum-Likelihood Phylogenies. Mol Biol Evol. 32:268–274. doi: 10.1093/molbev/msu300.

Podsiadlowski L et al. 2006. The mitochondrial genomes of *Campodea fragilis* and *Campodea lubbocki* (Hexapoda: Diplura): High genetic divergence in a morphologically uniform taxon. Gene. 381:49–61. doi: 10.1016/j.gene.2006.06.009.

Robinson KM, Sieber KB, Hotopp JCD. 2013. A Review of Bacteria-Animal Lateral Gene Transfer May Inform Our Understanding of Diseases like Cancer. PLOS Genet. 9:e1003877. doi: 10.1371/journal.pgen.1003877.

Stamatakis A. 2014. RAxML version 8: a tool for phylogenetic analysis and post-analysis of large phylogenies. Bioinformatics. 30:1312–1313. doi: 10.1093/bioinformatics/btu033.

Waterhouse RM et al. 2018. BUSCO Applications from Quality Assessments to Gene Prediction and Phylogenomics. Mol. Biol. Evol. 35:543–548. doi: 10.1093/molbev/msx319.

Yu G, Smith DK, Zhu H, Guan Y, Lam TT-Y. 2017. ggtree: an r package for visualization and annotation of phylogenetic trees with their covariates and other associated data. Methods Ecol. Evol. 8:28–36. doi: 10.1111/2041-210X.12628.
