# Supplementary figures and images for: The Genome of the Blind Soil-Dwelling and Ancestrally Wingless Dipluran Campodea augens: A Key Reference Hexapod for Studying the Emergence of Insect Innovations
Source: Genome Biol Evol. 2019 Dec 3;12(1):3534–49. doi: 10.1093/gbe/evz260 (PMC6938034; doi:10.1093/gbe/evz260)

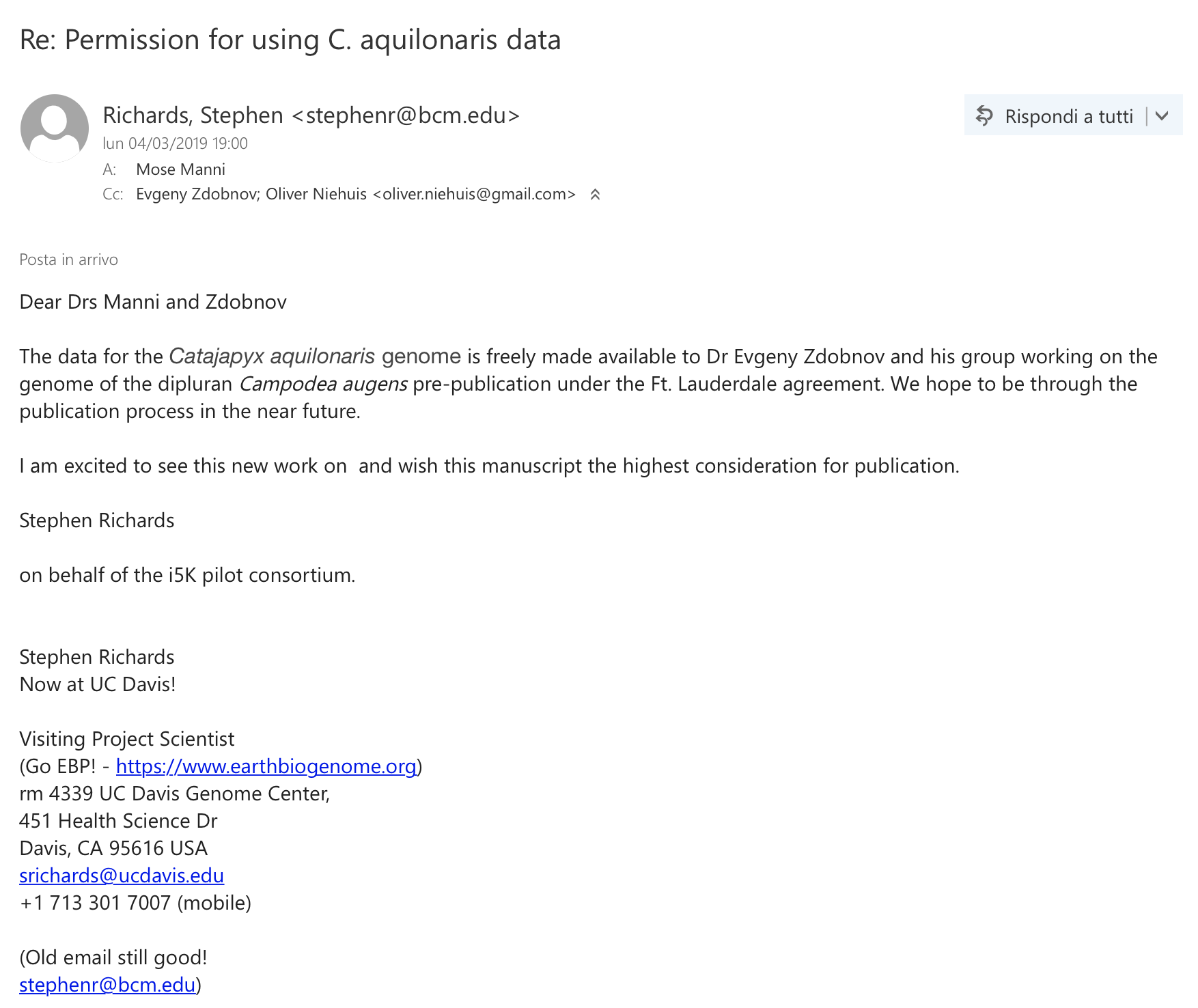

Supplement: evz260_Supplementary_Data [file evz260_supplementary_data.zip › Data usage permission C.aquilonaris.png]
